# *In Vitro* Anti-Influenza Virus Activities of a New Lignan Glycoside from the Latex of *Calotropis gigantea*

**DOI:** 10.1371/journal.pone.0104544

**Published:** 2014-08-07

**Authors:** Supawadee Parhira, Zi-Feng Yang, Guo-Yuan Zhu, Qiao-Lian Chen, Bei-Xian Zhou, Yu-Tao Wang, Liang Liu, Li-Ping Bai, Zhi-Hong Jiang

**Affiliations:** 1 State Key Laboratory of Quality Research in Chinese Medicine, Macau Institute for Applied Research in Medicine and Health, Macau University of Science and Technology, Taipa, Macau; 2 State Key Laboratory of Respiratory Disease, The First Affiliated Hospital of Guangzhou Medical University, Guangzhou, People's Republic of China; University of Hong Kong, Hong Kong

## Abstract

A new lignan glycoside, (+)-pinoresinol 4-*O*-[6″-*O*-vanilloyl]-*β*-d-glucopyranoside (**1**) and two known phenolic compounds, 6′-*O*-vanilloyltachioside (**2**) and 6′-*O*-vanilloylisotachioside (**3**) were isolated from the latex of *Calotropis gigantea* (Asclepiadaceae). The structure of the new compound was elucidated by using spectroscopic and chemical methods. Three isolates (**1–3**) and one authentic compound, (+)-pinoresinol 4-*O*-*β*-d-glucopyranoside, were screened for A/PR/8/34 (H1N1) inhibitory activity by cytopathic effect (CPE) inhibition assay on MDCK cells. Compound **1** showed inhibitory activity against A/PR/8/34 (H1N1). In sharp contrast, the other three compounds (**2**, **3** and (+)-pinoresinol 4-*O*-*β*-d-glucopyranoside) did not show such activity. An analysis of structure-activity relationship between **1** and (+)-pinoresinol 4-*O*-*β*-d-glucopyranoside revealed that the presence of a vanilloyl group in the sugar moiety of **1** is crucial for its anti-influenza virus activity. Compound **1** was further evaluated for *in vitro* inhibitory activities against a panel of human and avian influenza viruses by CPE inhibition assay. It showed inhibitory effect against human influenza viruses in both subtypes A and B (IC_50_ values around 13.4–39.8 µM with SI values of 3.7–11.4), while had no effect on avian influenza viruses. Its antiviral activity against human influenza viruses subtype A was further confirmed by plaque reduction assay. The time course assay indicated that **1** exerts its antiviral activity at the early stage of viral replication. A mechanistic study showed that **1** efficiently inhibited influenza virus-induced activation of NF-*κ*B pathway in a dose-dependent manner, but had no effect on virus-induced activation of Raf/MEK/ERK pathway. Further studies demonstrated that nuclear translocation of transcription factor NF-*κ*B induced by influenza virus was significantly blocked by **1**, meanwhile, nuclear export of viral ribonucleoproteins was also effectively inhibited. These findings suggest that this new lignan glycoside from *Calotropis gigantea*, may have therapeutic potential in influenza virus infection through inhibition of NF-*κ*B pathway and viral ribonucleoproteins nuclear export.

## Introduction

Influenza virus causes many upper respiratory tract infections. It is transmitted easily from person to person via airborne droplets and spreads rapidly in seasonal epidemics causing numerous people death from severe complications in pandemic years worldwide [1]. The latest global pandemic caused by H1N1 virus, characterized by a unique triple-reassortant gene segments of bird, swine and human influenza viruses, has resulted in more than 18,000 human deaths since it appeared in April 2009. More recently, the new avian-origin influenza A (H7N9) virus has caused 258 cases of human infection and 99 deaths in China as of April 2014 [1]–[3]. Moreover, the rate of emergence of viruses resistant to available antiviral drugs including M2 blockers (such as amantadine and rimantadine) and neuraminidase (NA) inhibitors (such as oseltamivir and zanamivir) has been increasing globally, which led to their effectiveness for viruses was greatly decreased [4], [5]. Therefore, it is urgently needed to develop safe and effective new antiviral drugs to combat viral infection either for therapeutic or prophylactic purposes. As such, many research groups have been trying to find new effective antiviral drug, especially, from the natural resources [6]–[9].

*Calotropis gigantea* (Asclepiadaceae) is a shrub common in Eastern Asia and Southeast Asia. The barks of this plant are used traditionally in Chinese folk medicine for the treatments of neurodermatitis and syphilis while the leaves are used as a poultice [10]. The latex of this plant has been used as an insecticidal agent [11], and as a purgative and a local irritant in indigenous medicine in India [12]. A fascinating series of bioactive secondary metabolites, e.g., cardiotonic steroids, triterpene alcohols, alkaloids and flavonoids have been isolated from different parts of *Calotropis* plants and showed various pharmacological properties such as analgesic, hepatoprotective, sedative, anti-inflammatory, anti-diarrheal, anti-asthmatic and anticancer activities [12]–[15]. In the present study, we successfully isolated a new lignan glycoside (**1**), together with two known phenolic compounds (**2** and **3**) from the latex of *C. gigantea* for the first time, and reported their *in vitro* anti-influenza virus activities and mechanism herein.

## Materials and Methods

### General experimental procedures

Optical rotation was measured on Rudolph Autopol I automatic polarimeter (USA). UV spectrum was performed on a Beckman Coulter DU800 spectrophotometer (USA). IR spectrum was obtained on a PerkinElmer Spectrum One Fourier transform infrared (FTIR) spectrometer (KBr). The ^1^H, ^13^C and 2D NMR experiments were conducted on a Bruker Ascend 600 NMR spectrometer (600 MHz for ^1^H and 150 MHz for ^13^C) by using the chemical shifts of NMR solvent, CD_3_OD, (^1^H, *δ*_H_ = 3.310; ^13^C, *δ*_C_ = 49.150) as the references. Chemical shifts are expressed in *δ* (ppm) and coupling constants (*J*) are given in Hz. ESI-TOF-MS spectra were obtained from an Agilent 6230 time-of-flight (TOF) mass spectrometer (USA). UHPLC analyses were carried out on an Agilent Technologies 1290 Infinity liquid chromatography system using an ACQUITY UPLC BEH C_18_ column (1.7 µm, 2.1×100 mm, Waters, Ireland). HPLC analysis was done on an Agilent 1100 series HPLC system using an Alltima C_18_ column (5 µm, 4.6×250 mm, Alltech, Grace, USA). Preparative high performance liquid chromatography was conducted on LabAlliance with VisionHT C_18_ Polar column (5 µm, 22×250 mm, Grace, USA). Medium Pressure Liquid Chromatography (MPLC, Sepacore, Buchi, Switzerland) was done using a Siliabond C_18_ ODS (40–63 µm, Silicycle, Canada) column (36×460 mm). Column chromatographies (CC) were performed using silica gel (75–150 µm, Accuchem, Czech Republic), silica gel 60 (50–55 µm, Merck, Germany), MCI-gel CHP 20P (75–150 µm, Mitsubishi Chemical Co. Ltd., Japan) and Bondapak Waters ODS (55–105 µm, Waters, USA). Thin-layer chromatography (TLC) was conducted on precoated HPTLC, TLC Kieselgel 60 F_254_ plates or TLC silica gel 60 RP-18 F_254s_ (200 µm thick, Merck KGaA, Germany). The spots were visualized under ultraviolet light (UV, wavelength 254 nm) and subsequently were sprayed by a solution of 5% sulfuric acid in 95% EtOH, followed by heating at 110°C. All stock solutions of tested compounds were prepared by dissolving in dimethyl sulfoxide (DMSO) in the concentration of 30 mM, and stored in −80°C. The desired concentration of each compound was diluted further by serum-free Minimum Essential Medium (MEM) with the final concentration of DMSO ≤1%.

### Chemicals and reagents

Absolute ethanol (AR grade) was purchased from Merck (Germany). Chloroform and ethyl acetate (AR grade) were bought from RCI Labscan Limited (Bangkok, Thailand). Methanol and acetronitrile (HPLC grade) were purchased from Tedia (USA). CD_3_OD was purchased from Sigma-Aldrich (USA). Sodium methoxide was a product of Sigma-Aldrich (USA). The purity of all isolated compounds for bioassay was more than 95% determined by UHPLC analysis.

### Plant material

The latex of *C. gigantea* was collected in Lampang, Thailand (latitude/longtitude at 17°36′9″N/99°12′50″E), during August–October 2011. The herbarium specimen was from a shrub which was authenticated by one of the authors, Dr. Li-Ping Bai. A voucher specimen (No. MUST-CG201011) was deposited at State Key Laboratory of Quality Research in Chinese Medicine, Macau University of Science and Technology. We state clearly that no specific permissions were required for these locations, because these locations are uncultivated land. We confirm that the field studies did not involve endangered or protected species.

### Extraction and isolation

The latex of *C. gigantea* (1 L) was added 95% EtOH to produce a filterable precipitate as described previously by Bhaskara and Seshadri to obtain ethanolic extract [11]. The mixture was sonicated at room temperature then centrifuged. The supernatant was evaporated under reduced pressure to afford a light yellowish residue (85.5 g). The residue was suspended in H_2_O then subjected to liquid-liquid partition by adding EtOAc. The residue (6.3 g) of EtOAc layer was subjected to silica gel CC (CHCl_3_–MeOH–H_2_O, 10∶0∶0 to 6∶4∶1) to obtain 4 fractions (Fr. 1 to 4). In the present study, two polar fractions (Fr. 3 and Fr. 4) were selected to do further purification. Fr. 3 (1.2 g) was chromatographed over MCI-gel CHP 20P CC (MeOH–H_2_O, 30∶70–100∶0) to afford 6 fractions (Fr. 3-1 to Fr. 3-6). Fr. 3-4 (300 mg) was loaded to Bondapak Waters ODS CC (MeOH–H_2_O, 50∶50 to 100∶0) to give 7 fractions (Fr. 3-4-1 to Fr. 3-4-7). Fr. 3-4-5 (76.8 mg) was purified by silica gel 60 CC (CHCl_3_–MeOH–H_2_O, 10∶0∶0 to 7∶3∶0.5) to afford 11 fractions (Fr. 3-4-5-1 to Fr. 3-4-5-11). Finally, Fr. 3-4-5-5 (20.1 mg) was further purified by MCI-gel CHP 20P CC (MeOH–H_2_O, 30∶70 to 100∶0) to yield compound **1** (5.0 mg).

Fr. 4 (0.7 g) was separated by MCI–gel CHP 20P CC (MeOH–H_2_O, 50∶50 to 100∶0) to afford 13 fractions (Fr. 4-1 to Fr. 4-13). Fr. 4-3 (67 mg) was subjected to silica gel 60 CC (CHCl_3_–MeOH–H_2_O, 10∶0∶0 to 7∶3∶0.5) to give 3 fractions (Fr. 4-1 to Fr. 4-3). Fr. 4-2 (8 mg) was further purified by preparative HPLC (35% isocratic aqueous MeOH) to afford compound **2** (2 mg) and **3** (3 mg).

To obtain compound **1** in larger amount, the latex of *C. gigantea* (3 L) was isolated with the similar protocols described previously by our group [15]. Briefly, three liters of the latex were precipitated by using 95% EtOH. The ethanolic extract was evaporated before subjected to liquid-liquid partition (H_2_O/EtOAc) for 3 times. The combined EtOAc layer was evaporated to dryness in vacuo to afford EtOAc residue. The residue of EtOAc layer (20.7 g) was further subjected to silica gel CC (CHCl_3_–MeOH–H_2_O, from 10∶0∶0 to 6∶4∶1) to obtain 8 fractions (Fr. A to H). In the present study, the polar fraction (Fr. G) was selected to do further purification because it was a compound **1**-abundant fraction detected by TLC and HPLC. Fr. G (3.0 g) was purified by MPLC [C_18_ Siliabond ODS, 20–80% MeOH in H_2_O] to obtain 7 fractions (Fr. G1 to Fr. G7). Fr. G6 (2.0 g) was resubjected to MPLC [C_18_ Siliabond ODS, 30–60% MeOH in H_2_O] to furnish 3 fractions (Fr. G6-1 to Fr. G6-3). Fr. G6-1 (659 mg) was finally purified by MPLC [C_18_ Siliabond ODS, 40–60% MeOH in H_2_O] to afford compound **1** (127 mg).

### Mild alkaline hydrolysis of compound 1

A solution of compound **1** (0.2 mg, 0.3 µmol) in MeOH (1 ml) was treated with sodium methoxide (NaOMe, 1.6 mg, 30 µmol) for 48 h at room temperature [16], [17]. The reaction was terminated by adding formic acid after two products of (+)-pinoresinol 4-*O*-*β*-d-glucopyranoside and a methyl ester of 4-hydroxy-3-methoxy benzoic acid were clearly detected by TLC analysis. The reaction mixture was then subjected to UHPLC-ESI-TOF-MS (positive mode) analysis to confirm reaction products of (+)-pinoresinol 4-*O*-*β*-d-glucopyranoside and a methyl ester of 4-hydroxy-3-methoxybenzoic acid. (+)-Pinoresinol 4-*O*-*β*-d-glucopyranoside was identified by comparing its UHPLC retention time (5.457 min) and ESI-TOF-MS data ([M+Na]^+^ at *m/z* 543.1841, calculated for C_26_H_32_O_11_Na, [M+Na]^+^ at *m/z* 543.1837) with those of an authenticated compound [18]. A methyl ester of 4-hydroxy-3-methoxybenzoic acid was identified by its accurate mass [M+H]^+^ at *m/z* 183.0650 (calculated for C_9_H_11_O_4_, [M+H]^+^ at *m/z* 183.0652).

### Compound 1

(+)-Pinoresinol 4-*O*-[6″-*O*-vanilloyl]-*β*-d-glucopyranoside, a light brown powder; [α]^25^_D_+16.48 (c 0.1, MeOH); IR (KBr): *ν*_max_ = cm^−1^ 3405, 1705, 1598 and 1515 cm^−1^ (Figure S1); UV (MeOH): λ_max_ nm (log ε) = 221 (4.60) and 266 (4.22) (Figure S2); ESI-TOF-MS (positive mode, Figure S3) found [M+Na]^+^ at *m/z* 693.2149 (calculated for C_34_H_38_O_14_Na at *m/z* 693.2154); ^1^H, ^13^C and 2D NMR (CD_3_OD) spectroscopic data (Figure S4, S5, S6, S7, S8), see Table 1.

**Table 1:** ^1^H (600 MHz) and ^13^C (150 MHz) NMR spectroscopic data (in CD_3_OD) of compound 1.

Moiety	Position	1			
		*δ*_C_	*δ*_H_(*J* in Hz)	HMBC (H to C)	COSY (H to H)
Aglycone moiety	1	137.3			
	2	111.6	6.97 *s*	1, 4, 6, 7	6
	3	150.6			
	4	147.1			
	5	117.6	6.98 *d* (8.4)	1, 3, 4	6
	6	119.2	6.46 *dd* (8.4, 1.8)	2, 4, 7	2, 5
	7	87.0	4.70 *d* (5.4)	1, 2, 6, 8, 9′	8
	8	55.4	3.01 *m*	1, 8′	7, 9, 8′
	9	72.8	4.27 *dd* (9.0, 7.2)	7, 8	8, 9
			3.83 *m*	8, 7′	8, 9
	3-OMe	56.7	3.86 *s*	3	
	1′	133.7			
	2′	111.0	7.00 *d* (1.8)	4′, 6′, 7′	6′
	3′	149.1			
	4′	147.3			
	5′	116.0	6.80 *d* (7.8)	1′, 3′	6′
	6′	120.2	6.85 *dd* (7.8, 1.8)	7, 2′, 4′, 7′	2′, 5′
	7′	87.6	4.69 *d* (5.4)	1′, 2′, 6′	8′
	8′	55.3	3.11 *m*	7, 8, 1′	8, 7′, 9′
	9′	72.5	4.15 *dd* (9.0, 6.6)	7′	8′, 9′
			3.87 *m*	7, 8′	8′, 9′
	3′ -OMe	56.4	3.90 *s*	3′	
Sugar moiety	1″	102.4	4.88 *d* (7.2)	4	2″
	2″	74.8	3.54 *m*	1″, 3″	1″, 3″
	3″	77.8	3.53 *m*	2″, 4″	2″, 4″
	4″	72.2	3.43 *t* (8.4)	3″, 5″	3″, 5″
	5″	75.6	3.79 *m*	4″, 6″	4″, 6″
	6″	65.0	4.65 *dd* (11.7, 1.8)	7″′	5″, 6″
			4.49 *dd* (11.7, 7.8)	5″, 7″′	5″, 6″
Vanilloyl moiety	1″′	122.5			
	2″′	113.8	7.56 *d* (1.8)	1″′, 3″′, 4″′, 6″′, 7″′	6″′
	3″′	148.8			
	4″′	153.1			
	5″′	116.0	6.90 *d* (8.4)	1″′, 3″′, 4″′	6″′
	6″′	125.3	7.61 *dd* (8.4,1.8)	2″′, 4″′, 7″′	2″′, 5″′
	7″′	167.7			
	3″′-OMe	56.5	3.86 *s*	3″′	

### Virus strains and cell lines

Influenza virus A/PR/8/34 (H1N1), A/FM/1/47 (H1N1) and a seasonal influenza A/Aichi/2/68 (H3N2) were purchased from the American Tissue Culture Collection (ATCC). Influenza virus B/Lee/1940 was isolated from routine clinical specimens. Several strains of avian influenza virus, including A/Duck/Guangdong/2009 (H6N2), A/Duck/Guangdong/1994 (H7N3) and A/Chicken/Guangdong/1996 (H9N2), were kind gifts from Dr. Chen Jianxin who is a professor of South China Agriculture University. The influenza viruses were propagated in the allantoic cavities of chicken eggs [19]. The virus titers were determined by 50% tissue culture infectious dose (TCID_50_) assay. The concentrations of the viral stocks were 10^5^ (A/PR/8/34), 10^5.5^ (A/FM/1/47), 10^3.5^ (A/Aichi/2/68), 10^4^ (B/Lee/1940), 10^4^ (A/Duck/Guangdong/2009), 10^6^ (A/Duck/Guangdong/1994) and 10^5.7^ (A/Chicken/Guangdong/1996) TCID_50_/mL.

MDCK (Madin Darby Canine Kidney) and A549 (human alveolar epithelial carcinoma) cells were purchased from the ATCC. The above two cell lines were grown in 25 cm^2^ cell culture flask (Corning Incorporated), then passaged into 96, 48 and 24 well cell culture clusters (Corning Incorporated) when used. MDCK and A549 were cultured in monolayer in Dulbecco's Modified Eagle's medium (DMEM) and Ham's F12 medium (ATCC), respectively, supplemented with 10% fetal bovine serum (FBS), penicillin (100 U/mL) and streptomycin (10 µg/mL), and incubated at 37°C under 5% CO_2_ in a humidified atmosphere.

### Cytotoxicity assay

MDCK cells were seeded into 96-well plates at density of 2×10^4^ cells/well (100 µL), and cultured to reach 90% confluence at 37°C under 5% CO_2_ for 24 h. The medium was replaced with that containing various concentrations of compound **1** (10–750 µM, 100 µL/well) or ribavirin (10–1500 µM, 100 µL/well) and the cells were further incubated at 37°C for 48 h. The 20 µL of MTT (3-(4,5-Dimethylthiazol-2-yl)-2,5-diphenyltetrazolium bromide) at a concentration of 5 mg/ml was added to each well, and continued to incubate for 4 h. Then, the medium was removed and formazan crystals were solubilized with DMSO. Absorbance was measured at 570 nm using a microplate reader [20]. The 50% toxic concentration (TC_50_) was calculated by Reed-Muench analysis [21]. The negative control was cells only and the blank control was the reagent used.

### Cytopathic effect (CPE) inhibition assay

MDCK cells (2×10^4^ cells/well, 100 µL) were seeded into 96-well plates and cultured at 37°C under 5% CO_2_ for 24 h. For the anti-influenza activity assay, MDCK cells were inoculated with serial influenza virus strains at 100-fold of the 50% tissue culture infection dose (100TCID_50_) at 37°C for 2 h, followed by removal of the medium and addition of 100 µL of the tested compounds with desired concentrations (5–300 µM) or ribavirin (5–400 µM) in serum-free Minimum Essential Medium (MEM) supplemented with 2 µg/ml of L-1-(tosyl-amido-2-phenyl) ethyl chloromethyl ketone (TPCK)-treated trypsin (Sigma). After incubation for 48 h at 37°C under 5% CO_2_, the CPE induced by the influenza virus was measured microscopically [22]. The concentration required for 50% inhibition (IC_50_) of the virus–induced CPE was calculated by the Reed-Muench analysis [21]. Each value was an average from three independent experiments. Selectivity index (SI) was calculated by the ratio of TC_50_/IC_50_ [6].

### Plaque reduction assay (PRA)

MDCK Cells (2×10^5^ cells/well) were seeded into 24-well culture plates and incubated overnight. The cells were washed with phosphate buffer saline (PBS) then incubated with viruses [including A/PR/8/34 (H1N1), A/FM/1/47 (H1N1) and A/Aichi/2/68 (H3N2)] diluted in serum-free MEM containing 100 U/mL of penicillin and 0.1 mg/mL of streptomycin for 2 h at 34°C at the multiplicities of infection (MOI = 0.01). After viral adsorption, cell monolayer was covered with overlay medium of compound **1** and further cultured at 34°C under 5% CO_2_ for 48 h. Then, the overlay medium was removed, and the cell monolayer was fixed with 10% formalin, stained with 1% crystal violet, and plaques were counted [23].

### Time course assay (time-of-addition)

MDCK cells (2×10^4^ cells/well) in 48-well plates were prepared, then infected with virus A/PR/8/34 (H1N1) (MOI = 0.02) for 2 h. After infection, the medium was discarded and cells were washed with PBS three times. Then, compound **1** (134.3 µM) or oseltamivir (6.4 µM) were added to cells at 0 h, 2 h, 4 h, 6 h, 8 h and 10 h after infection. The supernatants were harvested at 12 h post-infection and virus titers were determined by CPE and real time PCR assay [23].

### Western blotting

For viral infection, A549 cells (2×10^4^ cells/well) were washed with PBS and subsequently incubated with virus A/PR/8/34 (MOI = 0.1) diluted in PBS/BA (PBS supplemented with 0.2% BSA, 1 mM MgCl_2_, 0.9 mM CaCl_2_, 100 U/mL penicillin and 0.1 mg/mL streptomycin) for 30 min at 37°C. Then, the inoculum was aspirated and cells were incubated with Ham's F12 medium (containing 0.2% BSA, 1 mM MgCl_2_, 0.9 mM CaCl_2_, 100 U/mL penicillin and 0.1 mg/mL streptomycin) in the absence and presence of different concentrations (37.5–150 µM) of compound **1** or Bay11-7085 (10 µM) for 24 h at 37°C.

Cell lysis and Western blot were performed as previously described [24]. Briefly, cells were lysed on ice for 10 min by the lysis buffer of RIPA (pH 7.2 50 mM Tris-HCl, 0.15 M NaCl, 1% NP40, 0.1% SDS, 0.5% DOC, 1 mM PMSF, 25 mM MgCl_2_, and supplemented with a phosphatase inhibitor cocktail). The concentration of protein was determined by using the BCA Protein Assay Kit (Pierce). Equivalent amounts of protein were resolved by 10% SDS-PAGE. Subsequently, proteins were electro-transferred to a polyvinylidene fluoride (PVDF) membrane. The PVDF membrane was blocked with 5% BSA/TBST and then reacted with the indicated antibody in the same buffer with gentle agitation overnight at 4°C. Following three times washing, the PVDF membrane was further incubated with horseradish peroxidase (HRP)-conjugated secondary antibodies (Cell Signaling Technology) for 60 min. Antibody-protein complexes were detected by using a western lighting chemiluminescence system (Thermo Pierce).

### Indirect immunofluorescence microscopy

Nuclear translocation of NF-*κ*B and export of viral ribonucleoprotein (RNP) complexes were monitored by indirect immunofluorescence as described previously [25]. Briefly, A549 cells (2×10^4^ cells/well) were grown on glass coverslips inside a 48-well plate. When they reached 50% confluence, cells were infected with A/PR/8/34 (H1N1) at a MOI = 1. Two hours of post-infection, the inoculum was aspirated and medium/BA containing 1% DMSO or compound **1** was added. After 8 h, cells were washed with PBS and fixed with 4% PFA in PBS for 30 min at room temperature. Cells were permeabilized with 0.5% Triton X-100 in PBS for 15 min and blocked with 5% FBS in PBS for 20 min at 37°C. After blocking, cells were incubated with rabbit monoclonal antibody to phosho-NF-*κ*B p65 (Ser536) (93H1) (1∶100) (Cell Signaling Technology), mouse monoclonal antibody to viral NP (1∶1000) (Abcam) overnight at 4°C. After further wash, cells were incubated with FITC-labelled goat anti-rabbit IgG (1∶150) and FITC-labelled goat anti-mouse IgG (1∶150), respectively. Nuclei were stained using 4′,6-diamidino-2-phenylindole (DAPI, Roche). Fluorescence was visualized with a Zeiss Axiovert 135 fluorescence microscope.

### Statistical analysis

The results were expressed as means ± SD as indicated. The difference was considered statistically significant when the *p*-value was less than 0.05. Student's *t*-test was used for comparison between compound **1** treatment group and the virus group.

## Results

### Isolation, purification and structural elucidation of compound 1

**Figure 1:**
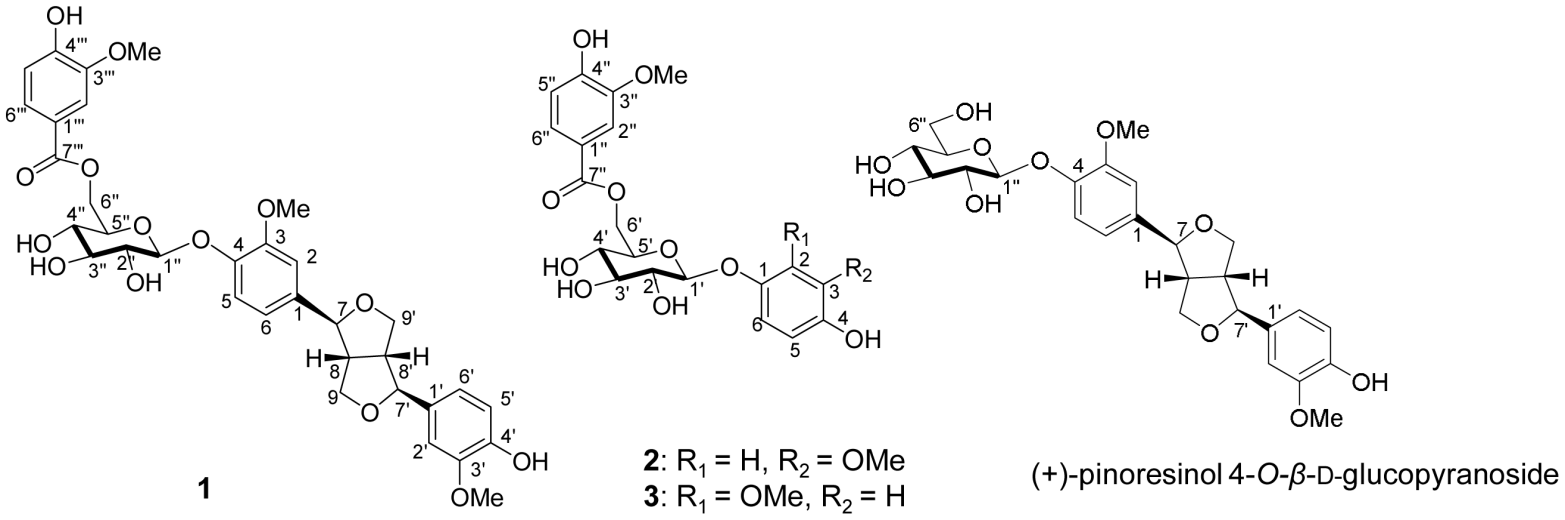
Chemical structures of compounds 1–3 and (+)-pinoresinol 4-*O*-*β*-d-glucopyranoside.

The latex of *C. gigantea* (1 L) was precipitated with 95% EtOH at the ratio of latex∶EtOH of 6∶4 at room temperature to give ethanolic solution and coagulum as described previously by Bhaskara and Seshadri [11]. The ethanolic solution was evaporated under reduced pressure before subjected to liquid–liquid partition by H_2_O–EtOAc. The EtOAc layer extract was subjected to a series of separation including normal and reverse phase open column chromatographies and preparative HPLC to afford compounds **1**, **2** and **3** (Figure 1). In order to obtain more compound **1**, the latex (3 L) was further purified by using beginning isolation procedures as described by our group [15]. The polar fraction (Fr. G) was selected to be further purified by repeated MPLCs to furnish compound **1** (127 mg).

Compound **1** was obtained as a light brown powder. Its molecular formula of C_34_H_38_O_14_ was established by the observation of [M+Na]^+^ at *m/z* 693.2149 (Figure S3) in the positive ESI-TOF-MS. The IR spectrum (Figure S1) indicated the existence of hydroxyl groups (3405 cm^−1^), carbonyl group (1705 cm^−1^) and aromatic rings (1598 and 1515 cm^−1^).

The ^1^H NMR spectrum of compound **1** (Table 1, Figure S4) displayed signals of three ABX benzene rings [26], an anomeric proton, three methoxyls, two oxygenated methylenes and two oxygenated methines. The ^13^C NMR spectrum (Figure S5) showed existence of three benzene rings, an ester carbonyl, a *β*-glucopyranosyl moiety and three methoxyl groups.

The presence of characteristic proton and carbon signals of two ABX benzene rings, two methoxy groups, two oxygenated methylenes, two oxygenated methines and a *β*-glucopyranosyl was closely resembled to those of (+)-pinoresinol 4-*O*-*β*-d-glucopyranoside [18], [27] except for the presence of proton and carbon signals for one more set of ABX benzene ring, a methoxy group and an ester carbonyl moiety which were in good agreement with the reported values of a vanilloyl moiety [28]. All these signals suggested the existence of moieties of (+)-pinoresinol 4-*O*-*β*-d-glucopyranoside and a vanilloyl group in compound **1**.

**Figure 2:**
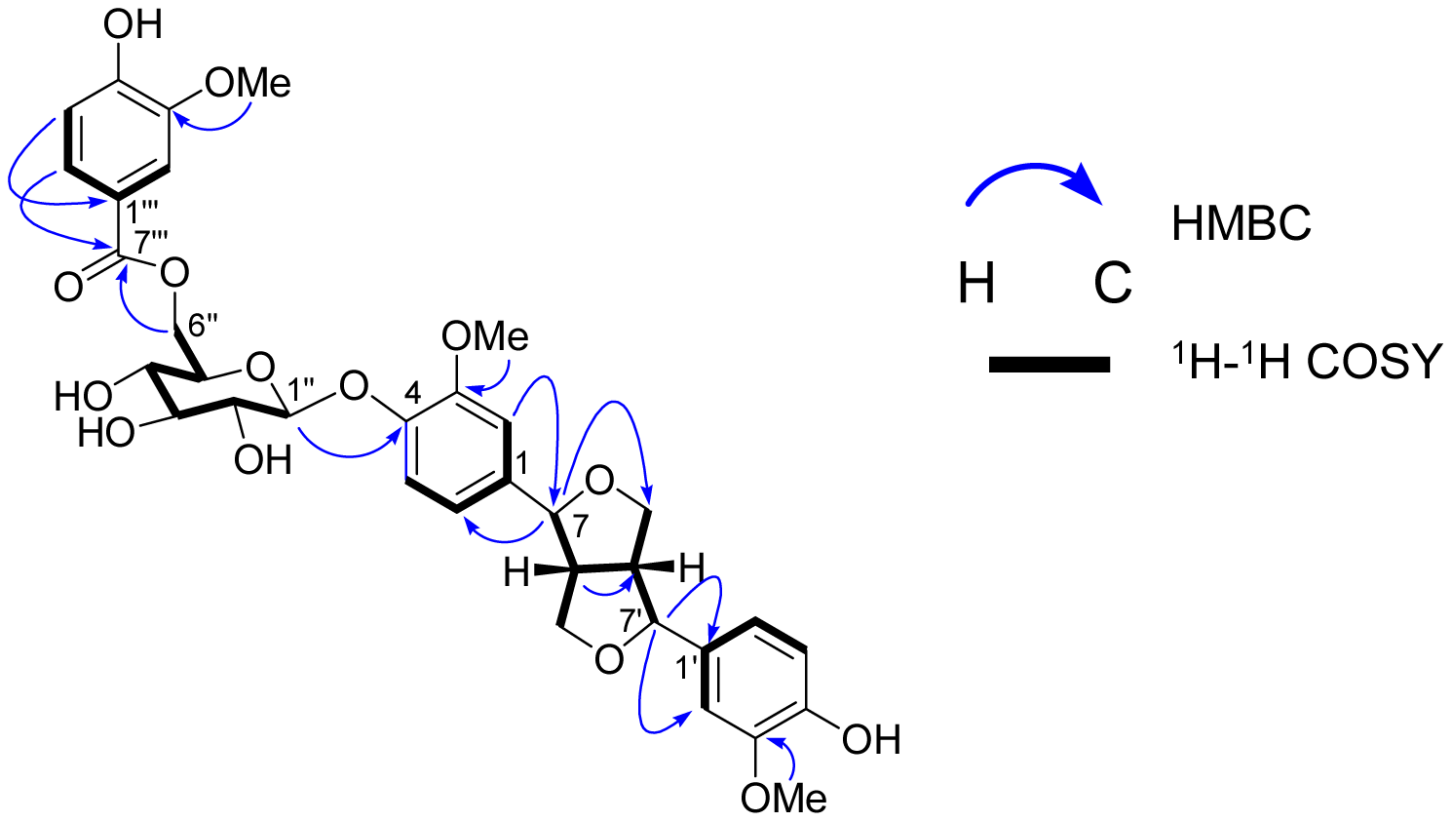
Selected HMBC and ^1^H–^1^H COSY correlations of compound 1. Arrows indicate the heteronuclear multiple-bond correlation of hydrogen atom to neighboring carbon. Bold line demonstrated the correlations between two nearby hydrogen atoms.

The ^1^H–^1^H COSY spectrum (Figure S8) exhibited the correlations (Figure 2) of typical proton signals of ABX benzene rings, i.e., the correlations of H-6 to H-2 and H-5 and H-6′ to H-2′ and H-5′, along with the correlations of characteristic resonances for two fused tetrahydrofuran moiety, i.e., the correlations of two aliphatic methines (H-8 and H-8′) to two oxygenated methines (H-7 and H-7′) and two oxygenated methylenes (H-9 and H-9′). Moreover, an anomeric proton (*δ*_H_ 4.65, *d*, *J* = 7.2 Hz, H-1″) of a glucopyranosyl group showed HMBC correlations (Figure S7) to C-4 (*δ_C_* 147.1), suggesting that the sugar part linked to the aglycone part at C-4 position. The large coupling constant at *J* = 7.2 Hz indicated the *β* anomeric configuration in glucopyranosyl moiety. These evidences strongly confirmed that the main skeletal part of compound **1** is (+)-pinoresinol 4-*O*-*β*-d-glucopyranoside. In addition, the correlations of H-6″′ to H-2″′ and H-5″′ in the ^1^H–^1^H COSY spectrum and HMBC correlation between H-6″′ (*δ*_H_ 7.61, *dd*, *J* = 8.4, 1.8 Hz) and an ester carbonyl at C-7″′ (*δ_C_* 167.7) confirmed the existence of a vanilloyl group in compound **1**.

**Figure 3:**
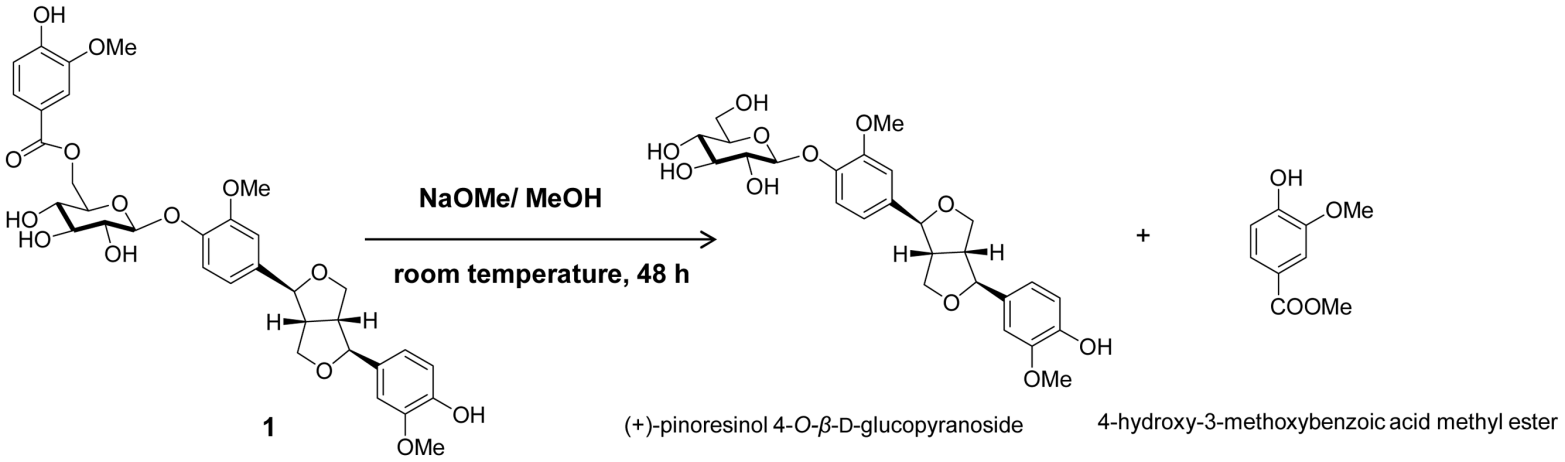
Mild alkaline hydrolysis of compound 1.

**Figure 4:**
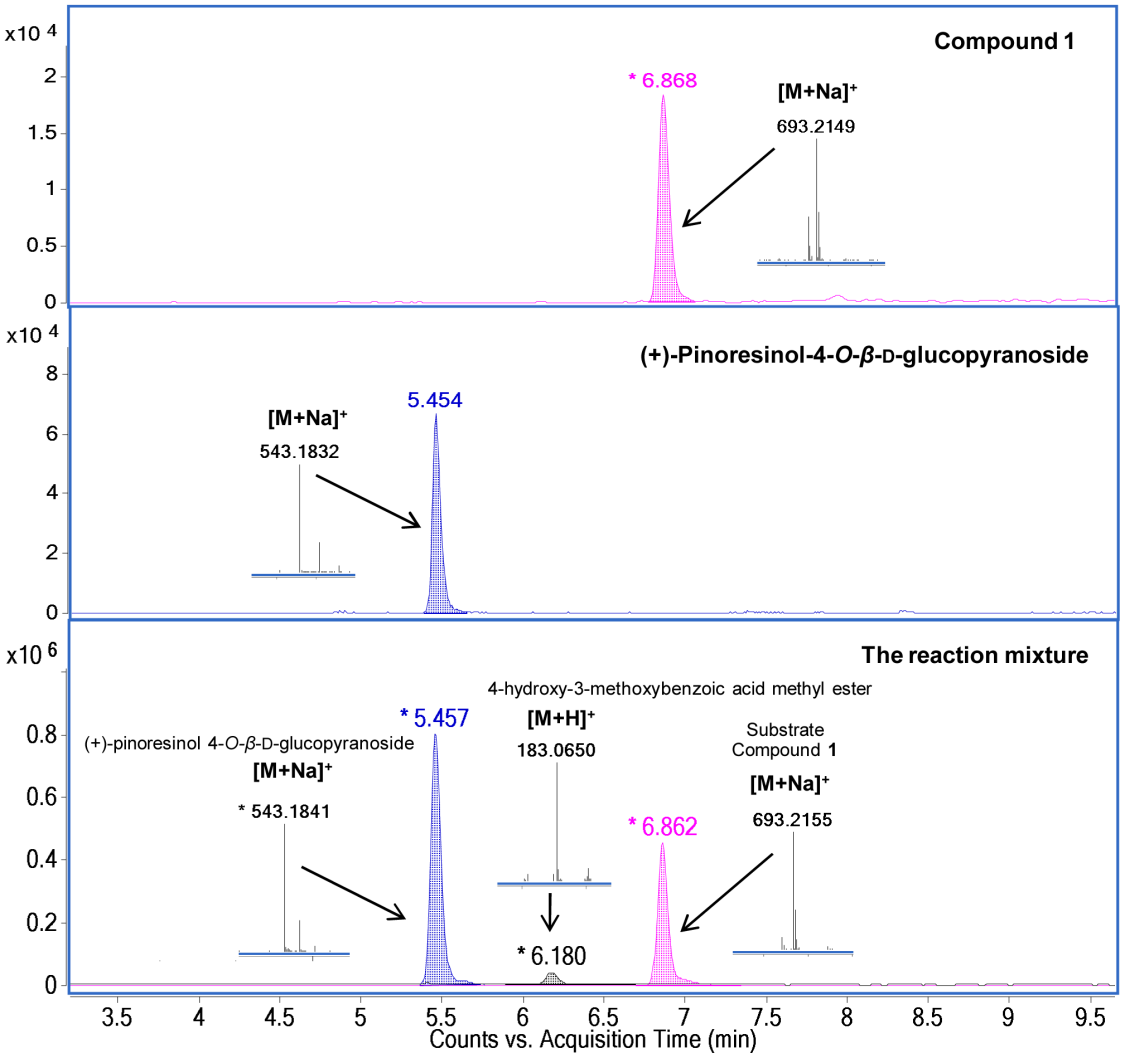
UHPLC-MS chromatograms and HRESIMS data of the products from alkaline hydrolysis of compound 1. The methanolic solution of compound **1** was treated with sodium methoxide to furnish (+)-pinoresinol 4-*O*-*β*-d-glucopyranoside and 4-hydroxy-3-methoxy benzoic acid methyl ester.

An alkaline hydrolysis (Figure 3) of compound **1** was performed to confirm the presence of (+)-pinoresinol 4-*O*-*β*-d-glucopyranoside moiety and a vanilloyl group in the molecule of compound **1**. One product of hydrolysis reaction of compound **1** was identified as (+)-pinoresinol 4-*O*-*β*-d glucopyranoside by comparison its retention time and MS data with those of authenticated compound [18], another one was characterized as a methyl ester of 4-hydroxy-3-methoxy benzoic acid by the presence of corresponding MS data at *m/z* 183.0654 [M+H]^+^ (Figure 4).

Furthermore, the evidence of downfield shift of C-6″ at glucopyranosyl moiety from *δ*_C_ 60.7 in (+)-pinoresinol 4-*O*-*β*-d-glucopyranoside [27] to *δ*_C_ 65.0 in compound **1** indicated the attachment of a vanilloyl group to C-6″ of sugar part. This linkage was further confirmed by the HMBC correlations from H-6″ (*δ*_H_ 4.65 and 4.49) to C-7″′ (*δ*_C_ 167.7) (Table 1, Figure 2). Therefore, the structure of compound **1** was assigned as (+)-pinoresinol 4-*O*-[6″-*O*-vanilloyl]- *β*-d-glucopyranoside.

### Identification of compounds 2 and 3

Two known compounds **2** and **3** have the same molecular formula of C_21_H_24_O_11_ by the evidences obtained from their positive ESI-TOF-MS of [M+Na]^+^ at *m/z* 475.1215 and 475.1217, respectively. Their ^1^H and ^13^C NMR data (Table 2, Figure S9, S10, S11, S12) showed the presence of a *β*-glucopyranosyl unit and a vanilloyl moiety which were in good agreement with those reported in literature [28]. Therefore, compounds **2** and **3** were characterized as 6′-*O*-vanilloyltachioside and 6′-*O*-vanilloylisotachioside, respectively.

**Table 2:** ^1^H (600 MHz) and ^13^C (150 MHz) NMR spectroscopic data (in CD_3_OD) of compounds 2 and 3.

Moiety	Position	2		3	
		*δ*_C_	*δ*_H_(*J* in Hz)	*δ*_C_	*δ*_H_(*J* in Hz)
Aglycone moiety	1	152.7		141.0	
	2	104.2	6.70 *s*	152.3	
	3	149.1^a^		101.9	6.43 *d* (2.7)
	4	143.3		155.1	
	5	116.0^b^	6.56 *d* (8.8)	107.6	6.11 *dd* (8.7, 2.7)
	6	110.2	6.55 *d* (7.4)	120.7	6.93 *d* (8.7)
	3-OMe	56.6^c^	3.70 *s*	56.6	3.78 *s*
Sugar moiety	1′	103.8	4.76 *d* (7.6)	104.3	4.71 *d* (7.6)
	2′	75.1	3.45-3.50 *m*	75.2	3.46-3.48 *m*
	3′	78.0	3.45-3.50 *m*	77.9	3.46-3.48 *m*
	4′	72.2	3.45-3.50 *m*	72.2	3.41 *m*
	5′	75.8	3.73 *m*	75.8	3.66 *m*
	6′	65.3	4.70 *dd* (11.8, 1.9)	65.2	4.66 *dd* (11.7, 2.1)
			4.37 *dd* (11.8, 7.4)		4.36 *dd* (11.7, 7.4)
Vanilloyl moiety	1″	122.1		122.5	
	2″	113.7	7.53 *d* (1.7)	113.8	7.53 *d* (1.8)
	3″	149.3^a^		148.9	
	4″	153.9		152.3	
	5″	116.3^b^	6.85 *d* (8.3)	116.1	6.86 *d* (8.2)
	6″	125.5	7.57 *dd* (8.3, 1.8)	125.4	7.55 *dd* (8.3, 1.8)
	7″	168.1		168.0	
	3″-OMe	56.4^c^	3.85 *s*	56.6	3.86 *s*

a to c Assignments may be interchanged within each column.

### Screening of *in vitro* anti-influenza virus activity of compounds 1–3 and (+)-pinoresinol 4-*O*-*β*-d-glucopyranoside against A/PR/8/34 (H1N1)

Four compounds (**1**, **2**, **3** and (+)-pinoresinol 4-*O*-*β*-d-glucopyranoside) were firstly evaluated for their antiviral activities towards an influenza virus strain A/PR/8/34 (H1N1) by CPE inhibition assay on MDCK cells. In the mode of treatment, compound **1** showed inhibitory effect on A/PR/8/34 (H1N1) virus-induced CPE in non-toxic concentration with an IC_50_ of 18.7 µM and a selectivity index (SI) larger than 11.3 (Table S1). While, the other three tested compounds did not exhibit any significant anti-H1N1 activity (SI<1). Except for a substitution of a vanilloyl moiety at C-6″ of compound **1** (Figure 1), the chemical structure of compound **1** closely resembles to (+)-pinoresinol 4-*O*-*β*-d-glucopyranoside. But their anti-H1N1 activities were notably different. This indicated that a vanilloyl group at C-6″ is an indispensable part for anti-H1N1 activity of compound **1**.

### *In vitro* anti-influenza virus activities of compound 1 against other influenza viruses

**Figure 5:**
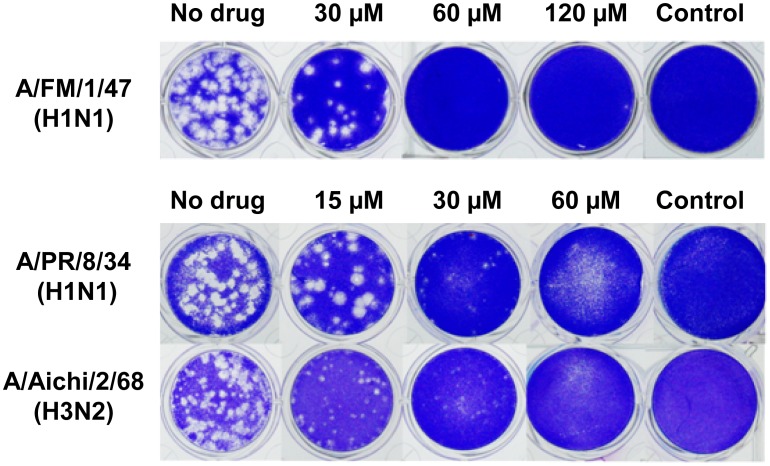
Plaque reduction assay of compound 1 against influenza viruses. MDCK cells were infected with influenza viruses, including A/FM/1/47 (H1N1) (Top row), A/PR8/8/34 (H1N1) (Middle row) and A/Aichi2/68 (H3N2) (Bottom row) at 0.01 MOI for 2 h at 34°C. After viral adsorption, cell monolayer was covered with overlay medium containing compound **1** and further cultured at 34°C under 5% CO_2_ for 48 h. Then, the overlay medium was removed, and the cell monolayer was fixed with 10% formalin, stained with 1% crystal violet, and plaques were counted.

The anti-influenza effect of compound **1** was further examined by using CPE assay against a series of both human and avian influenza viruses. As shown in Table 3, compound **1** exhibited antiviral activities at different magnitudes against influenza viruses from human isolates including influenza A [A/PR/8/34 (H1N1), A/FM/1/47 (H1N1) and A/Aichi/2/68 (H3N2)] and influenza B (B/Lee/1940) subtypes with IC_50_ values ranged from 13.4 to 39.8 µM and SI values of 3.7–11.4. The inhibitory effects of compound **1** against A/Aichi/2/68 (H3N2) and B/Lee/1940 were stronger (5.3 and 6.9 folds, respectively) than those of antiviral drug ribavirin, a positive control, while showed similar efficacy towards A/PR/8/34 (H1N1) and less inhibitory activity against A/FM/1/47 (H1N1) (Table 3). On the contrary, compound **1** displayed no effect towards three avian influenza viruses, including A/Duck/Guangdong/2009 (H6N2), A/Duck/Guangdong/1994 (H7N3) and A/Chicken/Guangdong/1996 (H9N2), with IC_50_ values >138.1 µM and SI<1 in all cases. The specific inhibitory effect on human influenza viruses of compound **1** was clearly demonstrated. The anti-influenza virus activities of compound **1** against A/PR/8/34 (H1N1), A/FM/1/47 (H1N1) and A/Aichi/2/68 (H3N2) were further examined by plaque reduction assay (PRA) (Figure 5). The IC_50_ values of compound **1** were in the range of 19.0–29.8 µM, with SI values of 4.6–7.3 (Table 3). The results were consistent with those of CPE assay.

**Table 3:** Antiviral activity of compound 1 against influenza viruses.

Virus type and strain	IC_50_ (µM)			SI		
	1*	1#	Ribavirin*	1*	1#	Ribavirin*
A/PR/8/34 (H1N1)	24.5±4.5	19.0±1.2	21.9±1.6	5.8±1.1	7.3±0.5	47.0±3.4
A/FM/1/47 (H1N1)	39.8±11.5	25.7±0.3	23.3±2.1	3.7±0.9	5.4±0.1	44.4±4.1
A/Aichi/2/68 (H3N2)	16.3±2.3	29.8±0.1	86.6±5.0	8.6±1.3	4.6±0.0	11.9±0.7
A/Duck/Guangdong/2009 (H6N2)	>138.1		81.5±3.9	<1.0		12.6±0.6
A/Duck/Guangdong/1994 (H7N3)	>138.1		63.8±4.6	<1.0		16.1±1.2
A/Chicken/Guangdong/1996 (H9N2)	>138.1		42.3±2.2	<1.0		24.3±1.3
B/Lee/1940	13.4±4.1		92.9±5.5	11.4±3.7		11.1±0.7

The concentration required for 50% cytotoxicity (TC_50_) of compound **1** and ribavirin is 138.1 µM and 1024.6 µM, respectively, to MDCK cells by MTT assay. Data obtained from CPE assay (*) and plaque reduction assay (#).

### Time course assay (time-of-addition) of compound 1 in influenza virus-infected cells

**Figure 6:**
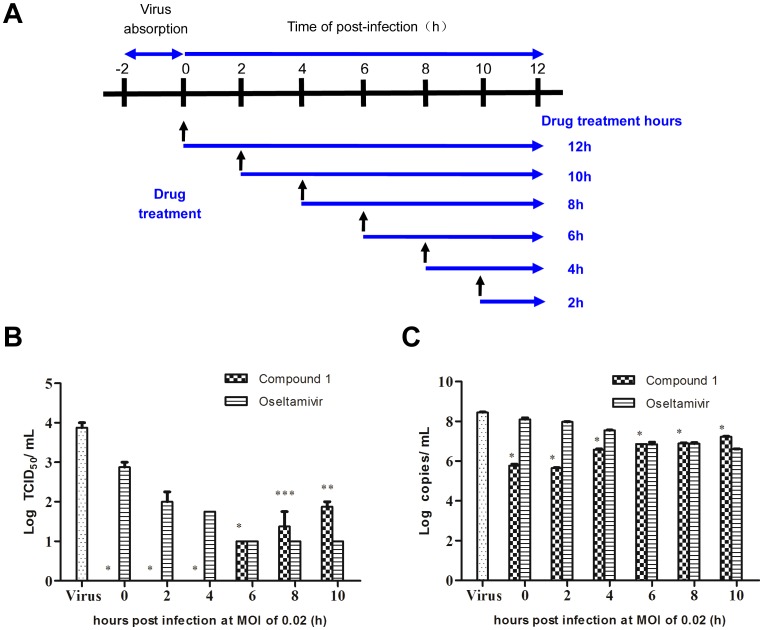
Time course assay of compound 1. (A) An illustration scheme shows the time of addition of compound **1**. (B–C) MDCK cells (2×10^4^ cells/well) in 48-well plates were infected with influenza virus A/PR/8/34 (H1N1) (MOI = 0.02). At post-infection, the medium was discarded and cells were washed with PBS three times. Compound **1** (134.3 µM) or oseltamivir (6.4 µM) was added at 0 h, 2 h, 4 h, 6 h, 8 h and 10 h after infection. At 12 h post-infection, the supernatants were collected and infectious titers were determined by CPE assay (B) and real time PCR assay (C). Data represent mean ± SD of 3 biological samples. Statistical significance was assessed by comparison between compound **1**-treated group and virus control group by using student's *t*-test analysis (* *p*-value <0.001, ** *p*-value <0.01 and *** *p*-value <0.05).

The antiviral mechanism of compound **1** was examined by a time course assay (time of drug addition, Figure 6A) in a single infectious cycle using an A/PR/8/34 (H1N1) infection model. The results showed that the clinically used anti-influenza drug, oseltamivir, reduced virus titers at the late stage (6 to 10 h) by blocking the release of progeny virions (Figure 6B), while compound **1** exerted its antiviral effect at the early stage of virus replication (0 to 4 h). Virus titers in the supernatant were remarkably reduced by compound **1** treatment (Figure 6B). Meanwhile, significant reduction of virus copies further confirmed the inhibitory effect of compound **1** on influenza virus (Figure 6C). These suggested that the antiviral mechanism of compound **1** was via inhibition of the early stage of influenza virus replication rather than interfering with virus release step.

### Inhibition of influenza virus-induced NF-*κ*B activation in A549 cells by compound 1

**Figure 7:**
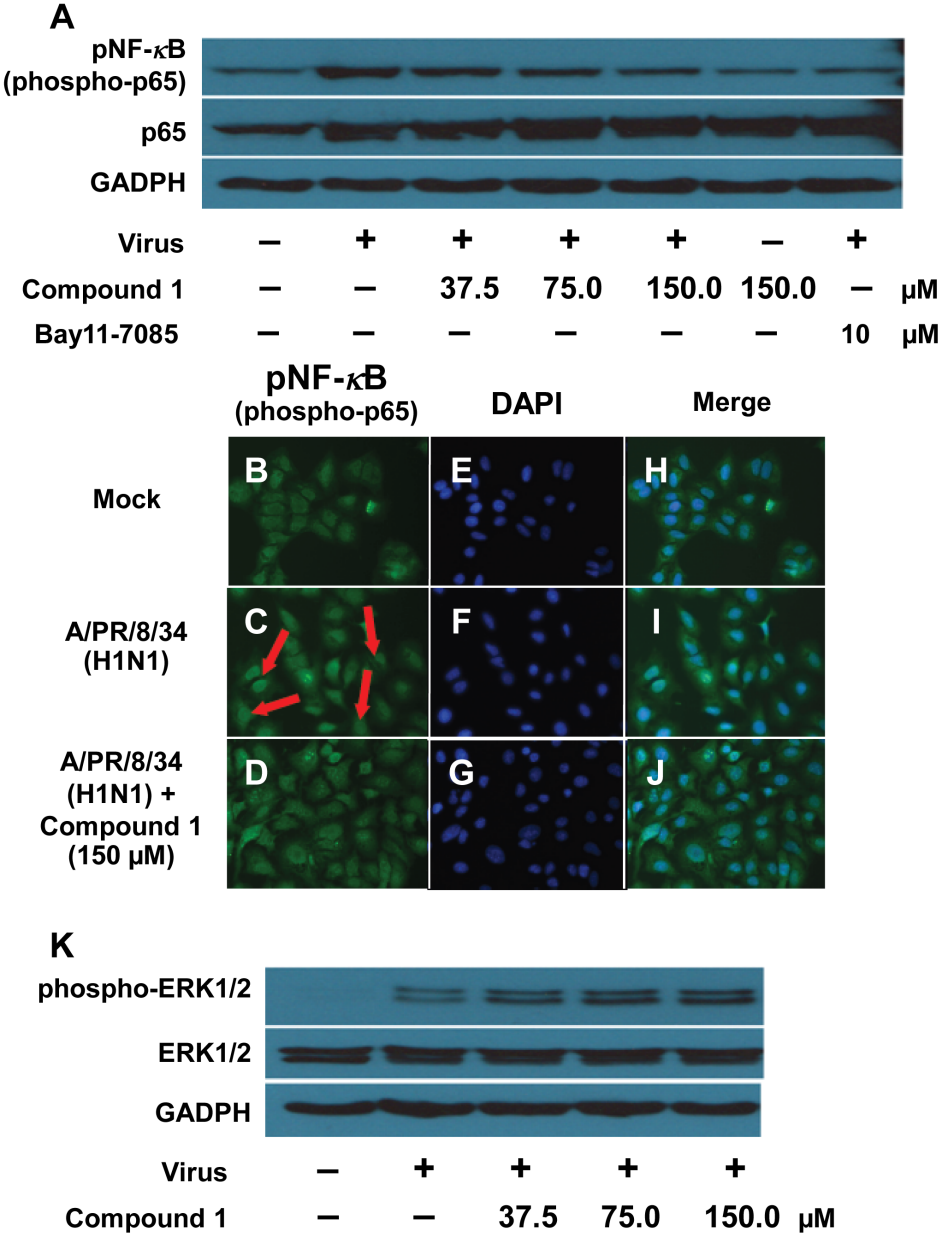
Inhibition of influenza virus-induced NF-*κ*B activation in A549 cells by compound 1. Compound **1** inhibited the influenza virus-induced NF- *κ*B activation (A–J) but did not affect virus-induced Raf/MEK/ERK pathway activation (K). (A) and (K): A549 cells were infected with A/PR/8/34 (H1N1) (MOI = 0.1) in the absence or presence of different concentrations of compound **1** or the specific NF-*κ*B inhibitor Bay11-7085 (10 µM) alone. A549 cell lysates were subjected to Western blot with specific antibodies against phospho-NF-*κ*B p65 (Ser536), total NF-*κ*B p65, phosphorylated ERK1/2 and ERK1/2. Equal protein load was verified using pan-antisera to GADPH. (B–J): A549 cells were infected with A/PR/8/34 (H1N1) (MOI = 1) and stained for against phospho-NF-*κ*B p65 (Ser536) at 10 h of post-infection (green). Cell nuclei were stained with DAPI (blue).

The activation of NF-*κ*B signaling pathway is a prerequisite for influenza virus infection [29]. The specific inhibitors of NF-*κ*B, such as Bay 11-7085, not only efficiently blocked replication of influenza viruses, but also reduced NF-*κ*B-regulated cytokines [25]. Here we showed that compound **1** inhibits virus-induced NF-*κ*B activation in a dose dependent manner (Figure 7A). To further confirm the inhibition of virus-induced NF-*κ*B activation by compound **1**, we utilized immunofluorescence to monitor nuclear translocation of the p65 subunit. We observed that compound **1** significantly blocked influenza virus-induced nuclear translocation of p65 (Figure 7B–J), which was consistent with its dose-dependent inhibitory effect on NF-*κ*B activation shown in Figure 7A. Activation of Raf/MEK/ERK signaling appears to support virus replication by regulating nuclear export of viral RNP [30]. However, we found that compound **1** did not affect virus-induced activation of the Raf/MEK/ERK pathway (Figure 7K). These results so far indicated that the antiviral effect of compound **1** is due to its NF-*κ*B-inhibiting activity.

### Compound 1 efficiently blocked nuclear export of viral RNPs in the infected A549 cells

**Figure 8:**
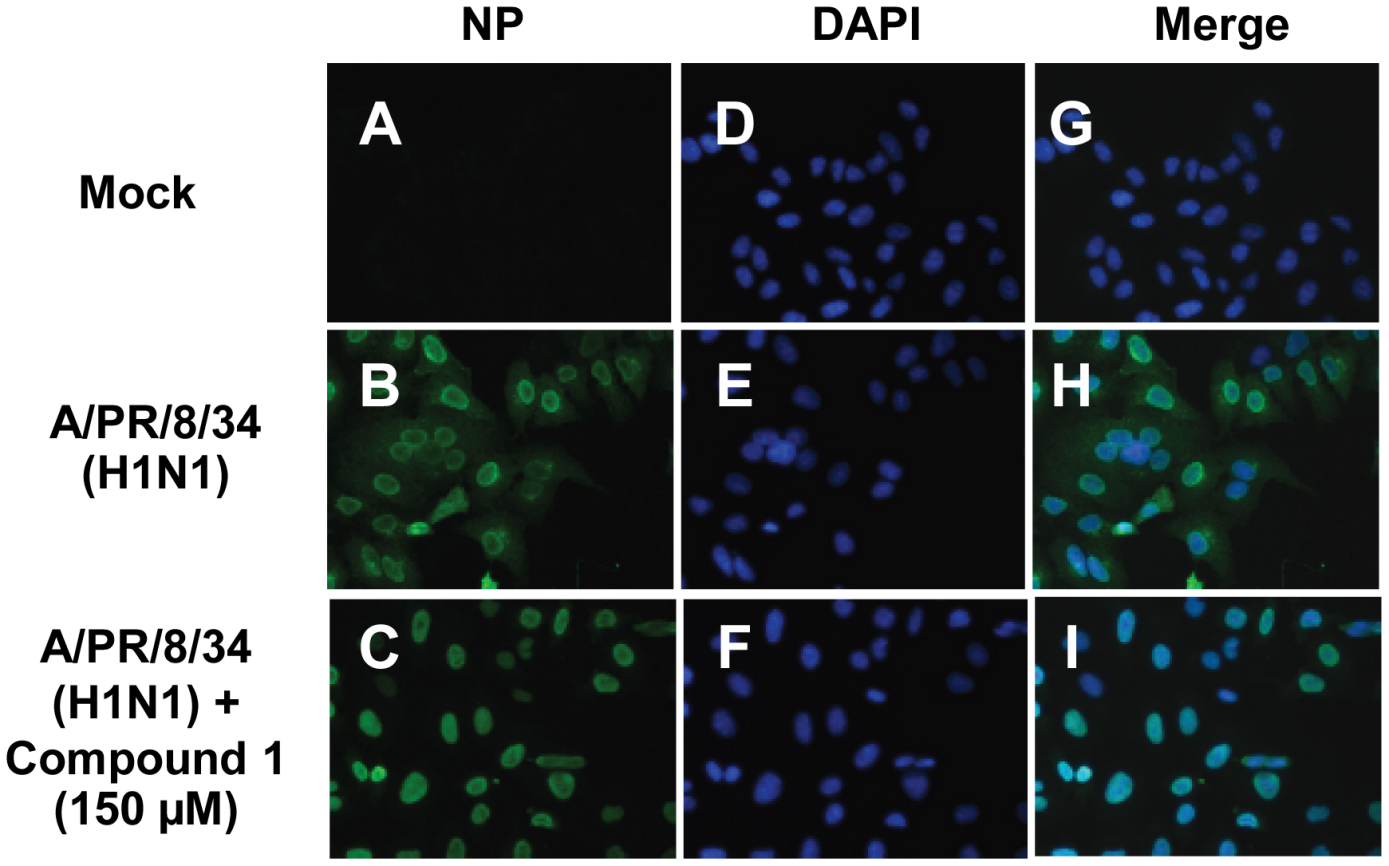
Effect of compound 1 on influenza virus-induced export of viral ribonucleoprotein complexes. Immunofluorescence confocal microscopy was used to show the nuclear RNP export in the absence or presence of compound **1**. A549 cells were infected with A/PR/8/34 (H1N1) (MOI = 1) and stained for influenza viral nucleoprotein (NP) at 10 h of post-infection (green). Cell nuclei were stained with DAPI (blue).

Nuclear retention of viral RNP complexes preventing formation of progeny virus particles would block virus propagation [31]. Recent reports demonstrated that viruses support NF-*κ*B-dependent expression of proapoptotic factor, FasL and TRAIL, which activate caspases that subsequently regulate nuclear export of the viral RNP complexes [29], [31]. Therefore, we investigated whether inhibiting NF-*κ*B activation by compound **1** could block nuclear export of viral RNP in the infected A549 cells. As a result, we observed that compound **1** treatment efficiently impaired nuclear export of the viral RNP in the infected A549 cells (Figure 8). This indicated that anti-influenza virus effect of compound **1** was predominantly through its NF-*κ*B-inhibiting activity to suppress viral RNP export and subsequent virus propagation.

## Discussion

Infection with influenza virus is still one of the severe threats worldwide, which caused significant morbidity and mortality. On March 30 in 2013, a novel multiple-reassortant avian influenza A subtype H7N9 infection was identified in eastern China [32]. The emergence of oseltamivir resistance of clinical isolates of H7N9 has been associated with Arg292Lys mutation in the virus neuraminidase (NA) gene [33]. Thus, there is an urgent need to explore and develop new anti-influenza drugs. Different parts of *C. gigantea* have exhibited a broad spectrum of pharmacological properties [12], [13], [15]. However, there has been a lack of studies regarding its effect on anti-influenza virus activity.

In contrast to two known phenolic compounds (**2** and **3**), the new lignan glycoside (**1**) exhibited a selective antiviral effect on human influenza viruses in both CPE and PRA assays. In a time-course assay, a notable and significant progeny virus reduction was observed when compound **1** was applied at an early phase of viral replication (0 to 4 h post-infection) (Figure 6B). Compared to the antiviral effect of oseltamivir, compound **1** exhibited less inhibitory activity at the late stage (6 to 10 h post-infection, Figure 6C). This implied that compound **1** efficiently blocks influenza viruses' entry or/and replication stages rather than release. Further investigations are required to clarify the specific target of compound **1** inhibiting influenza viruses' entry or/and replication.

Like all other viral pathogens, influenza virus utilizes the host cellular machinery to support their replication. Although NF-*κ*B plays an important role in the maintenance of host defense responses [34], independent studies demonstrated that the pathway is critical for efficient replication of influenza virus. Several distinct mechanisms have been proposed, including induction of NF-*κ*B-dependent proapoptotic factors, such as TRAIL and Fas/FasL, which involve in caspases-mediated nuclear export of viral RNP complexes [29], [31] or suppress antiviral response of type I IFN signaling through up-regulation of SOCS-3 [35] or by direct suppression of ISG promoter regions [36]. Thus, targeting of cellular signaling pathways that is essential for virus replication might be a promising antiviral strategy. The well-known NF-*κ*B inhibitor, acetylsalicylic acid (ASA), efficiently blocks influenza virus replication *in vitro* and *in vivo* [37]. Specific inhibitor, Bay 11-7085 that blocks NF-*κ*B activation, not only effectively reduced virus titers, but also decreased virus-induced pro-inflammatory cytokine and chemokine production [25]. In the present study, we have identified the inhibition of NF-*κ*B pathway by compound **1** in a dose-dependent manner (Figure 7A). Further immunofluorescence study confirmed compound **1** blocks influenza virus-induced nuclear translocation of p65 (Figure 7B–J). We also demonstrated that compound **1** impaired the nuclear export of viral RNP (Figure 8), which is an essential step to be packaged into virion particles. These results indicated that compound **1** suppresses influenza virus replication in a mechanism via its NF-*κ*B-inhibiting activity. Our findings are consistent with the previous studies that inhibition of NF-*κ*B activation resulted in impaired nuclear RNP export and therefore blocked influenza virus replication. This was also supported by the time-course assay that compound **1** targeted the early phase events of cellular NF-*κ*B activation for virus replication (Figure 6). Further study is required to explore the detailed mechanism by which compound **1** exerts inhibitory effect on influenza replication and nuclear export of viral RNP.

## Conclusions

A novel lignan glycoside, (+)-pinoresinol 4-*O*-[6″-*O*-vanilloyl]-*β*-d-glucopyranoside (**1**) and two known phenolic compounds, 6′-*O*-vanilloyltachioside (**2**) and 6′-*O*-vanilloylisotachioside (**3**) were isolated from the latex of *C. gigantea* for the first time. The structure of new compound was established by using both spectroscopic methods and chemical reaction. Three isolates and an authentic (+)-pinoresinol 4-*O*-*β*-d-glucopyranoside were screened for their anti-influenza activities by using CPE assay. It was found that compound **1** exhibited anti-influenza activity, while the other three compounds did not show such activity against an influenza virus strain A/PR/8/34 (H1N1). Discrepancy in anti-influenza viruses activity between compound **1** and (+)-pinoresinol 4-*O*-*β*-d-glucopyranoside, revealed the necessity of a vanilloyl moiety in compound **1** for its anti-influenza activity. Further investigation of its anti-influenza viral spectrum by CPE assay and PRA indicated that compound **1** possesses specific antiviral activity against a panel of human influenza viruses [A/PR/8/34 (H1N1), A/FM/1/47 (H1N1) and A/Aichi/2/68 (H3N2)] while shows no inhibitory activity towards avian influenza viruses. A preliminary mechanistic study showed that compound **1** suppresses an early stage of viral replication. Compound **1** inhibited A/PR/8/34 virus-induced phosphorylation of p65 in a dose-dependent manner, and significantly suppressed the translocation of the phosphorylated p65 (pNF-*κ*B) into the nucleus, indicating that compound **1** possesses inhibitory effect on the virus-induced activation of NF-*κ*B. In addition, compound **1** blocked the nuclear export of the viral RNP, which was irrelevant to the virus-induced activation of Raf/MEK/ERK signaling pathway. It was concluded that the anti-influenza virus activity of compound **1** closely correlates with its NF-*κ*B-inhibiting activity. Our studies suggested that this novel lignan glycoside (**1**) might be a promising candidate for the research and development of antiviral agents specifically targeting human influenza viruses.

## Supporting Information

Figure S1 **The IR spectrum of compound 1.**
(TIF)

Figure S2 **UV-VIS absorption spectrum of compound 1.**
(TIF)

Figure S3 **ESI-TOF-MS spectrum of compound 1.**
(TIF)

Figure S4 **^1^H NMR (600 MHz) spectrum of compound 1 in CD_3_OD.**
(TIF)

Figure S5 **^13^C NMR (150 MHz) spectrum of compound 1 in CD_3_OD.**
(TIF)

Figure S6 **HSQC spectrum of compound 1 in CD_3_OD.**
(TIF)

Figure S7 **HMBC spectrum of compound 1 in CD_3_OD.**
(TIF)

Figure S8 **^1^H–^1^H COSY of compound 1 in CD_3_OD.**
(TIF)

Figure S9 **^1^H NMR (600 MHz) spectrum of compound 2 in CD_3_OD.**
(TIF)

Figure S10 **^13^C NMR (150 MHz) spectrum of compound 2 in CD_3_OD.**
(TIF)

Figure S11 **^1^H NMR (600 MHz) spectrum of compound 3 in CD_3_OD.**
(TIF)

Figure S12 **^13^C NMR (150 MHz) spectrum of compound 3 in CD_3_OD.**
(TIF)

Table S1 **Screening of compounds 1–3 and (+)-pinoresinol 4-** ***O*** **-** ***β*** **-d-glucopyranoside for inhibiting influenza viruses [A/PR/8/34 (H1N1)] activity.**
(TIF)
