# Supplementary figures and images for: In Vitro Anti-Influenza Virus Activities of a New Lignan Glycoside from the Latex of Calotropis gigantea
Source: PLoS One. 2014 Aug 7;9(8):e104544. doi: 10.1371/journal.pone.0104544 (PMC4125211; doi:10.1371/journal.pone.0104544)

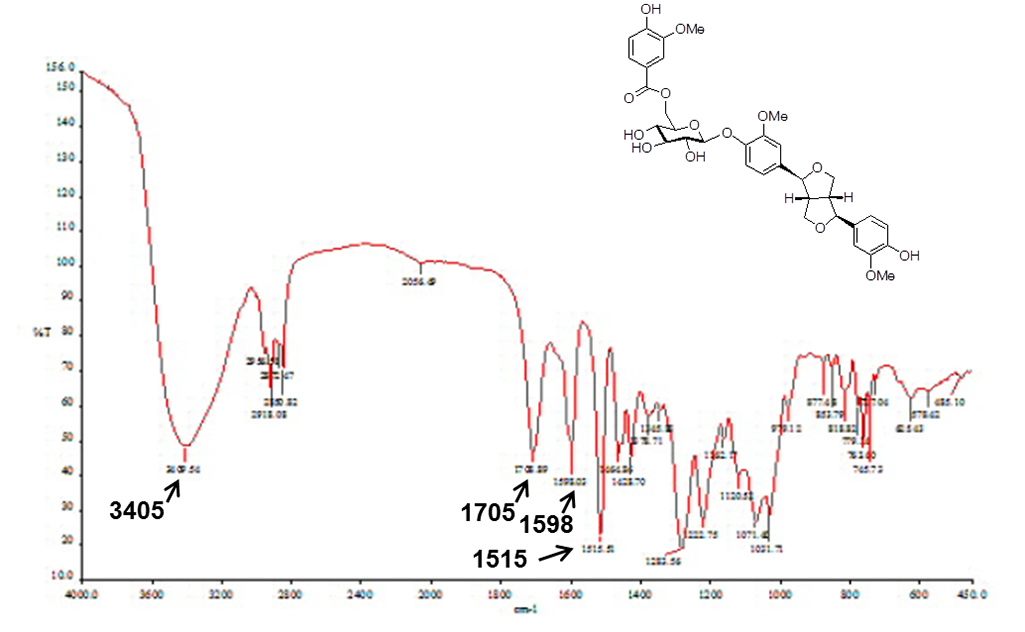

Supplement: Figure S1 — The IR spectrum of compound 1. (TIF) [file pone.0104544.s001.tif]

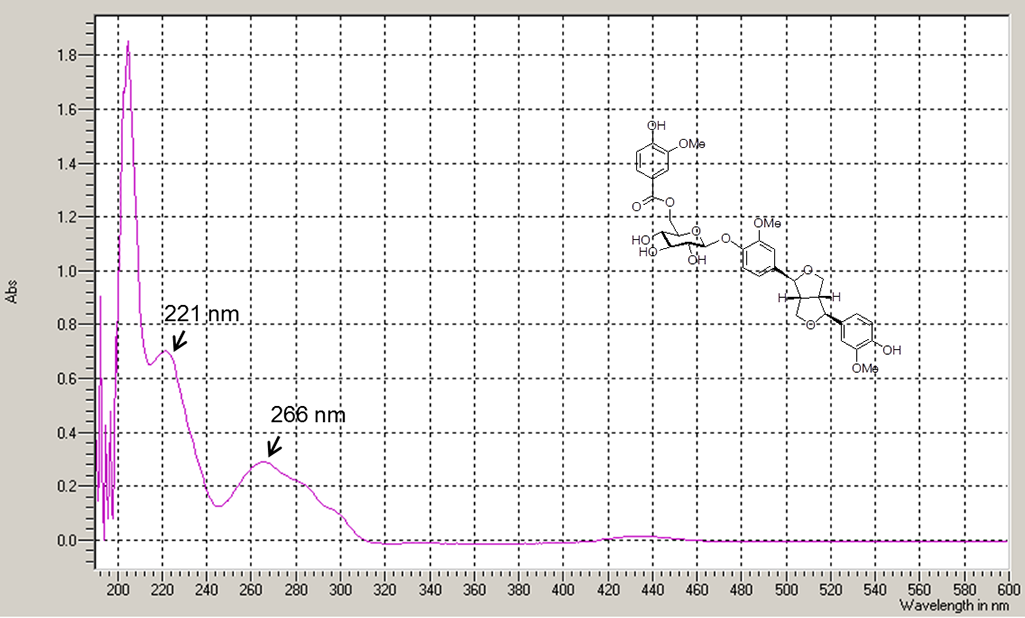

Supplement: Figure S2 — UV-VIS absorption spectrum of compound 1. (TIF) [file pone.0104544.s002.tif]

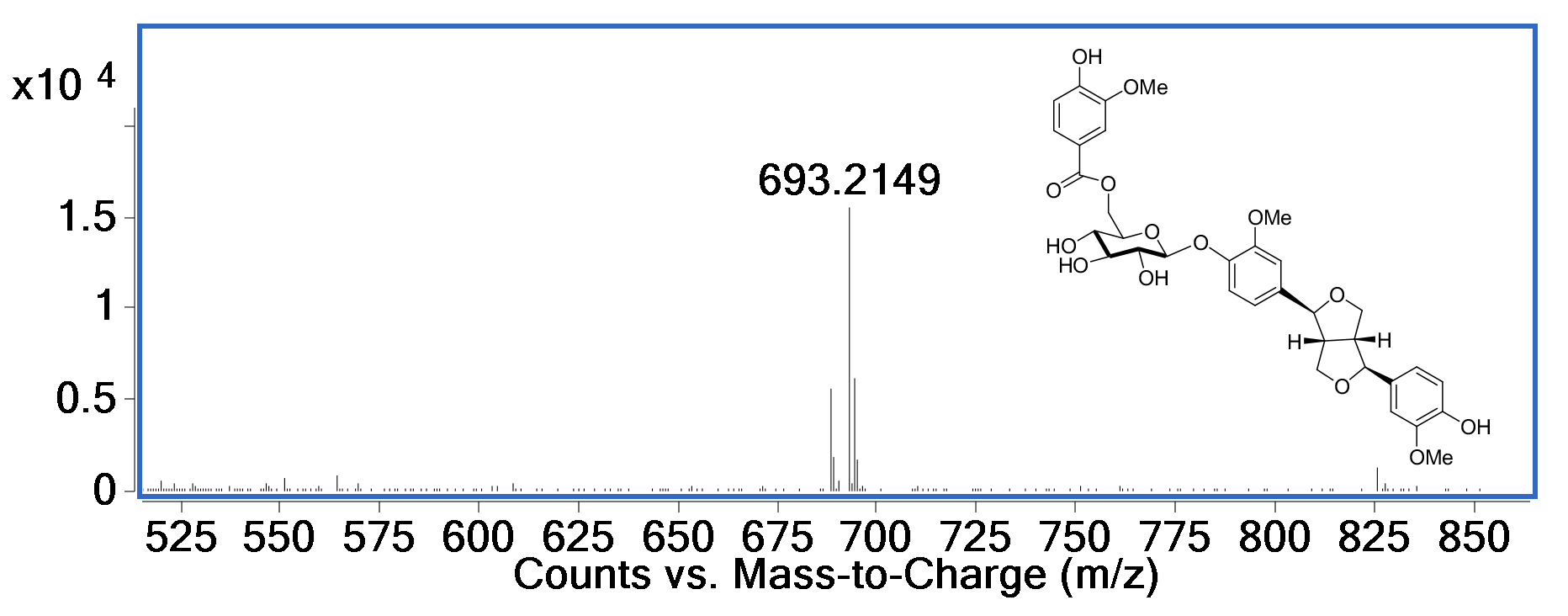

Supplement: Figure S3 — ESI-TOF-MS spectrum of compound 1. (TIF) [file pone.0104544.s003.tif]

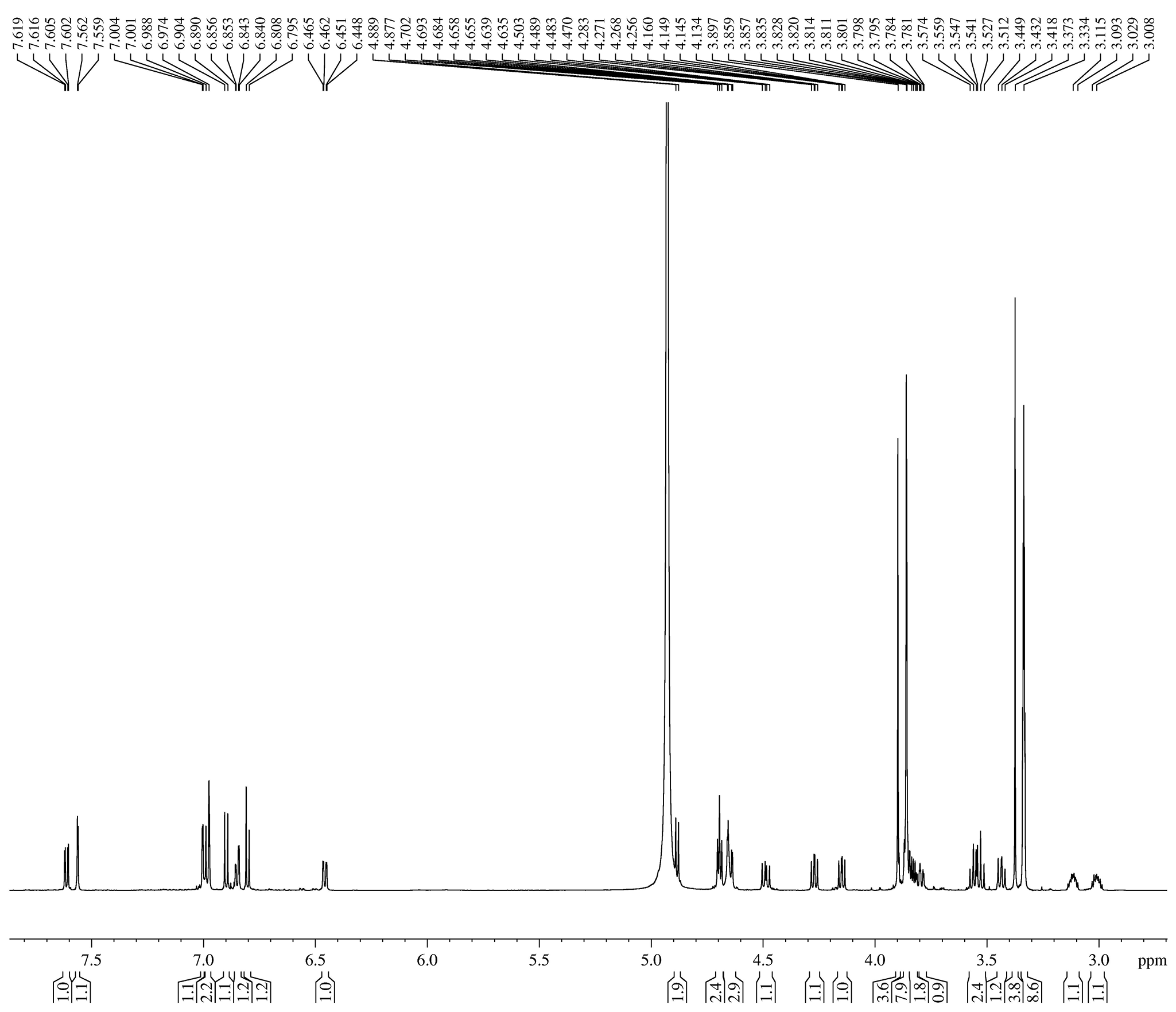

Supplement: Figure S4 — 1H NMR (600 MHz) spectrum of compound 1 in CD3OD. (TIF) [file pone.0104544.s004.tif]

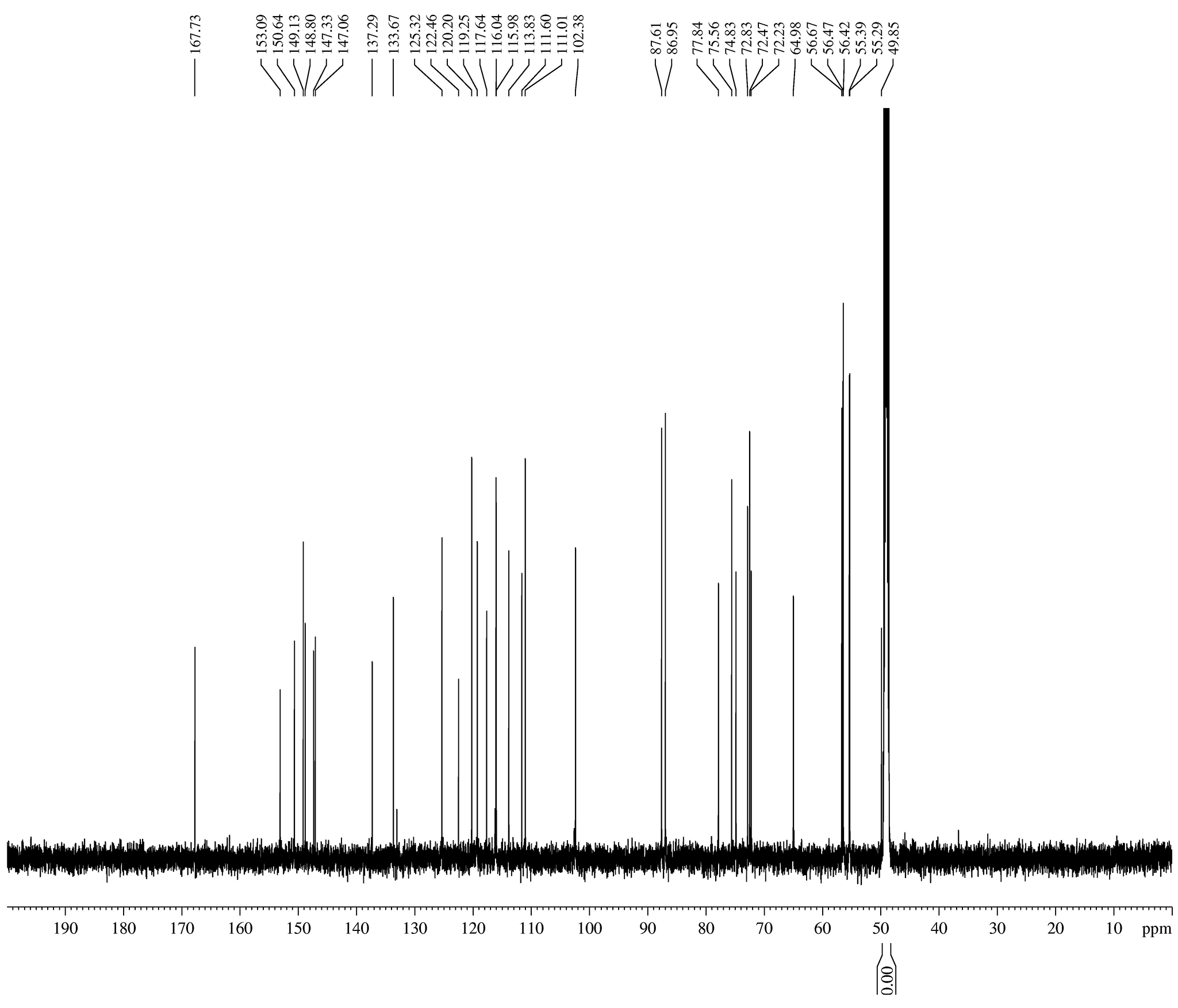

Supplement: Figure S5 — 13C NMR (150 MHz) spectrum of compound 1 in CD3OD. (TIF) [file pone.0104544.s005.tif]

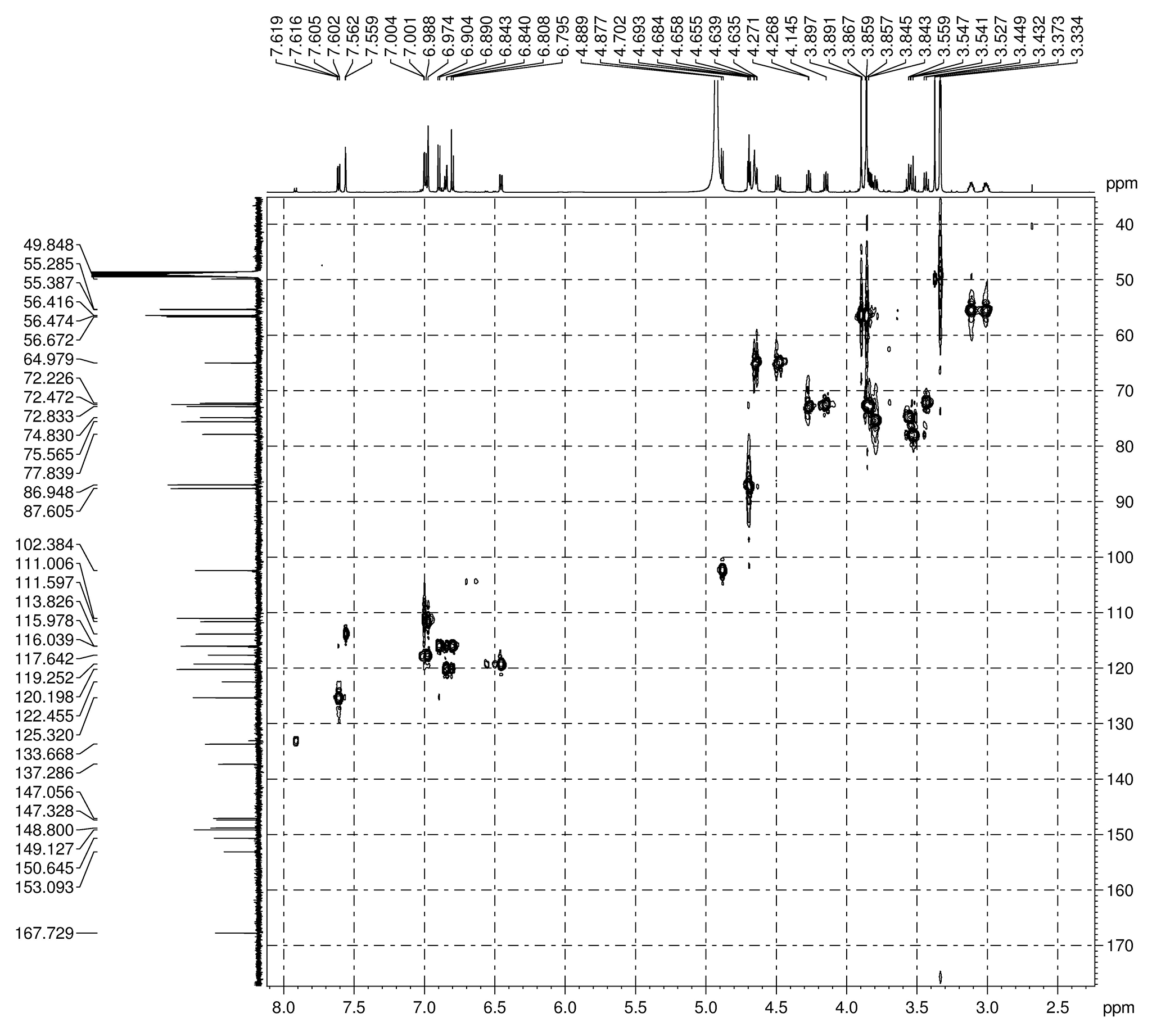

Supplement: Figure S6 — HSQC spectrum of compound 1 in CD3OD. (TIF) [file pone.0104544.s006.tif]

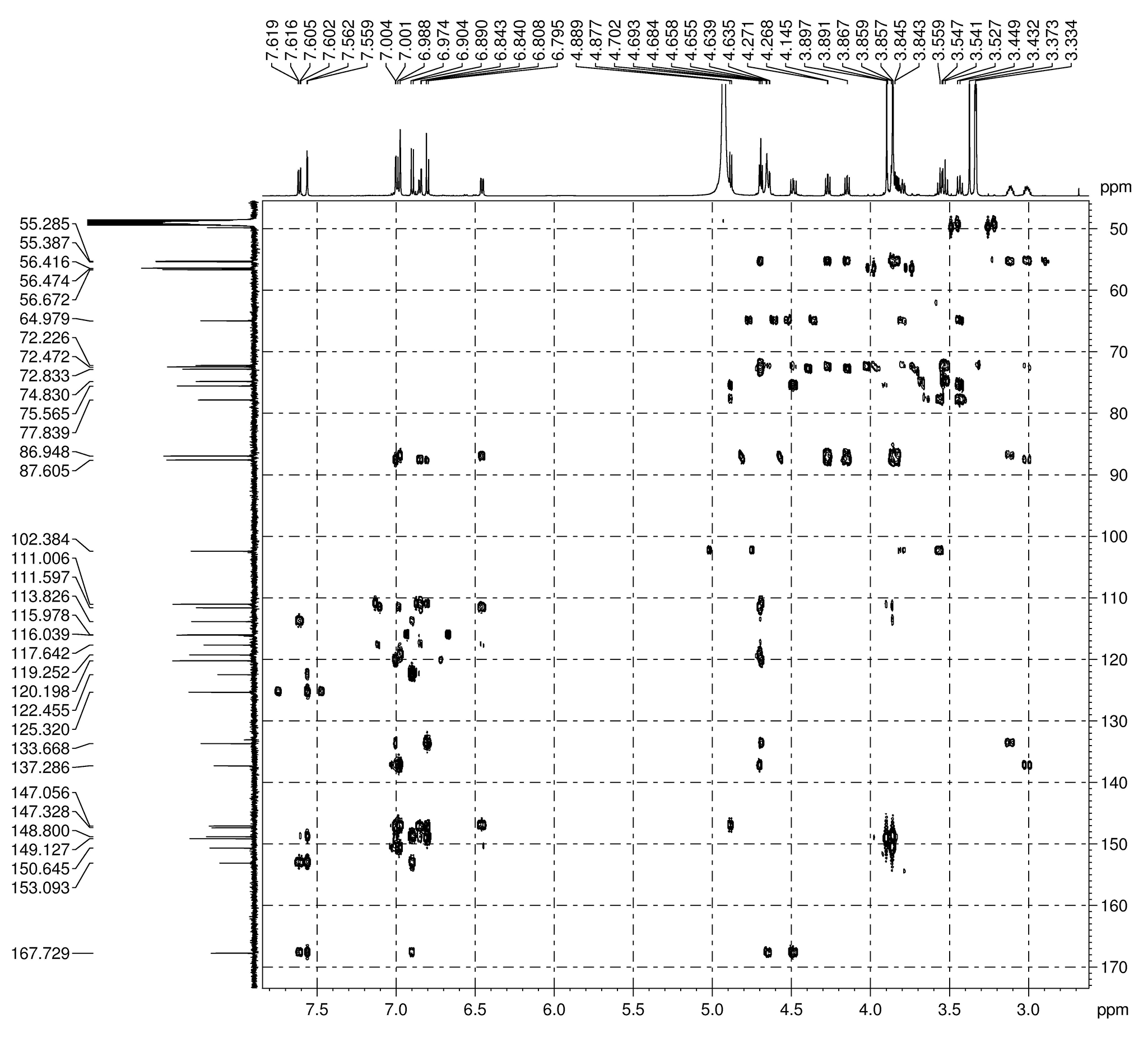

Supplement: Figure S7 — HMBC spectrum of compound 1 in CD3OD. (TIF) [file pone.0104544.s007.tif]

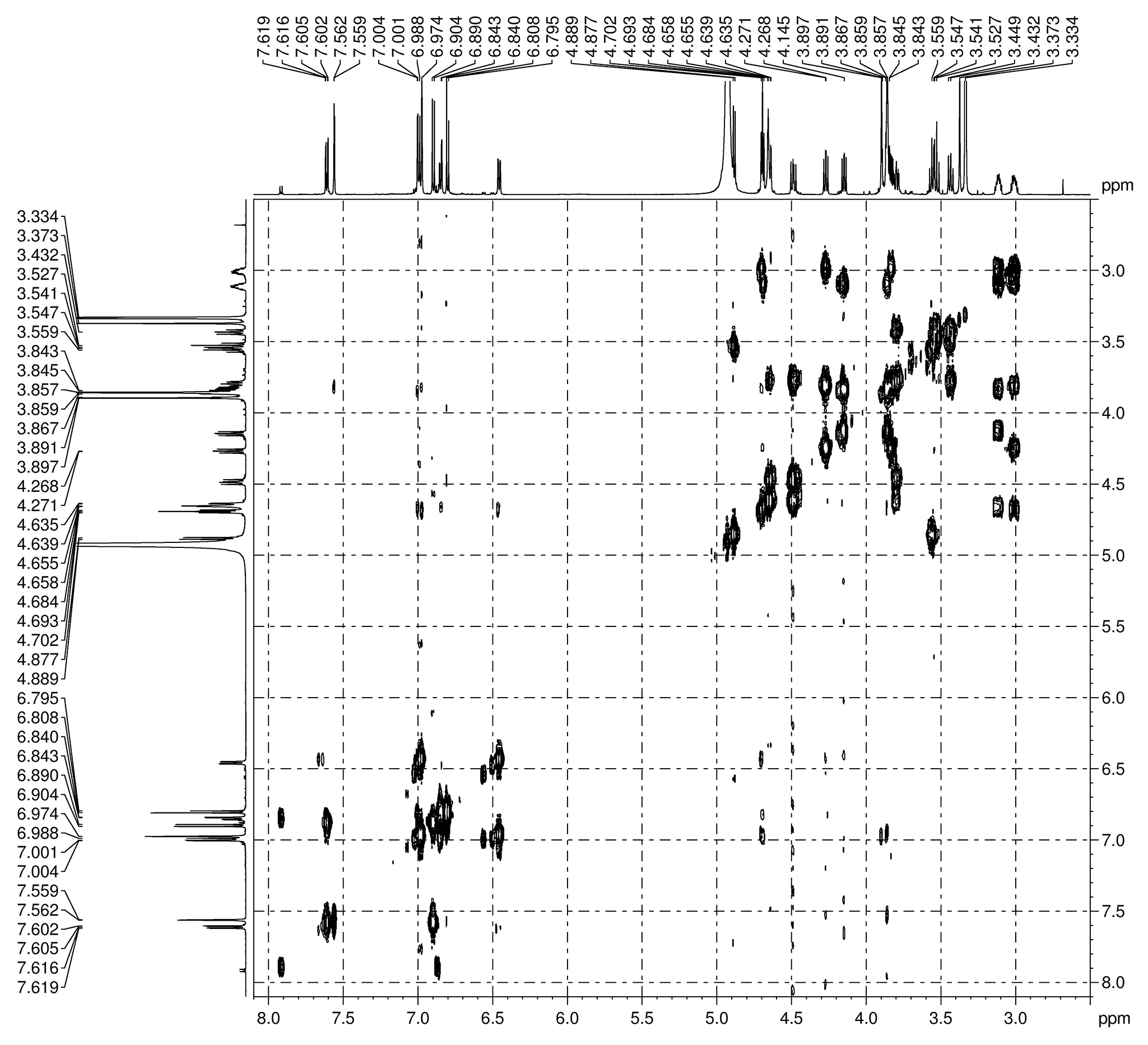

Supplement: Figure S8 — 1H–1H COSY of compound 1 in CD3OD. (TIF) [file pone.0104544.s008.tif]

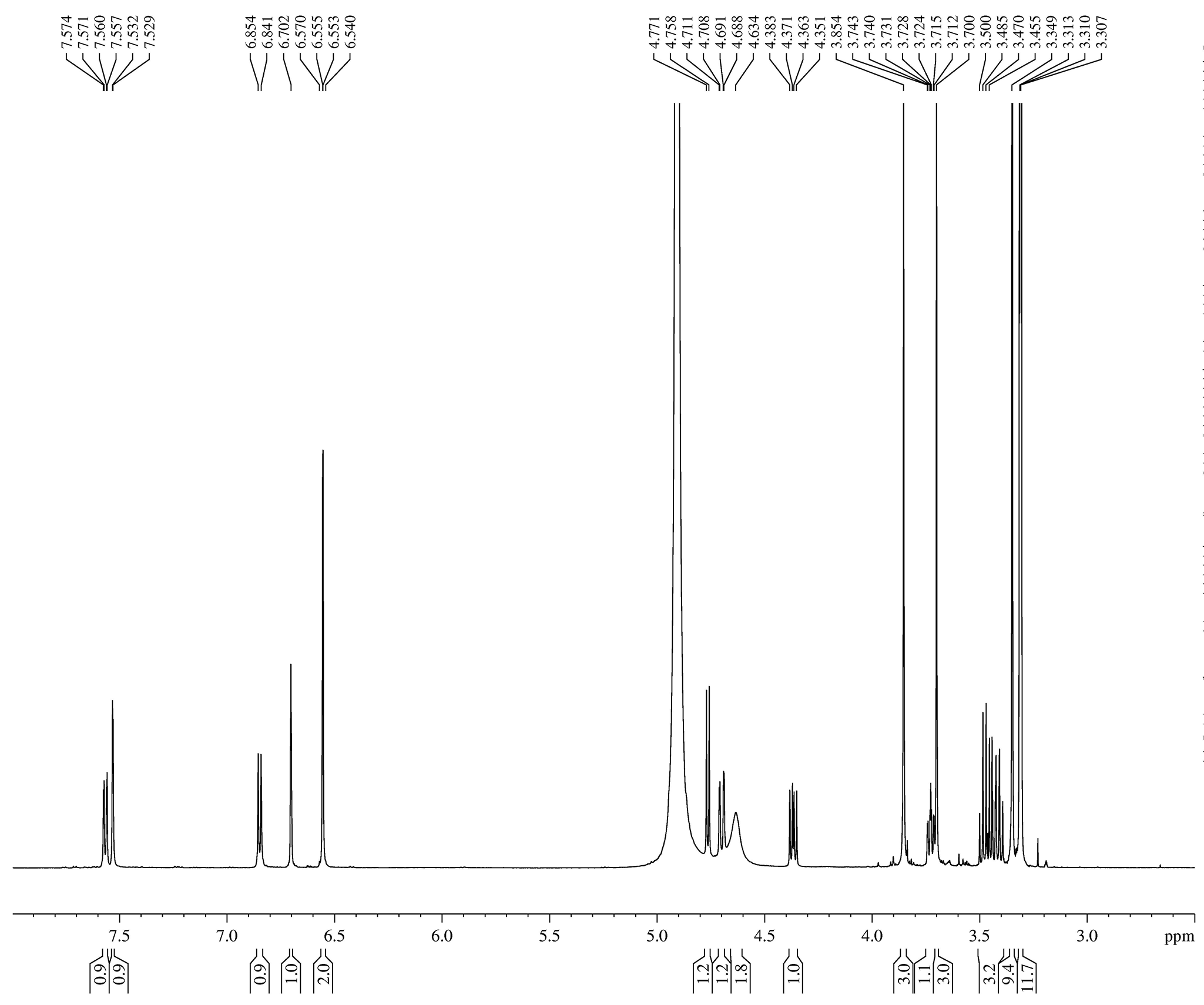

Supplement: Figure S9 — 1H NMR (600 MHz) spectrum of compound 2 in CD3OD. (TIF) [file pone.0104544.s009.tif]

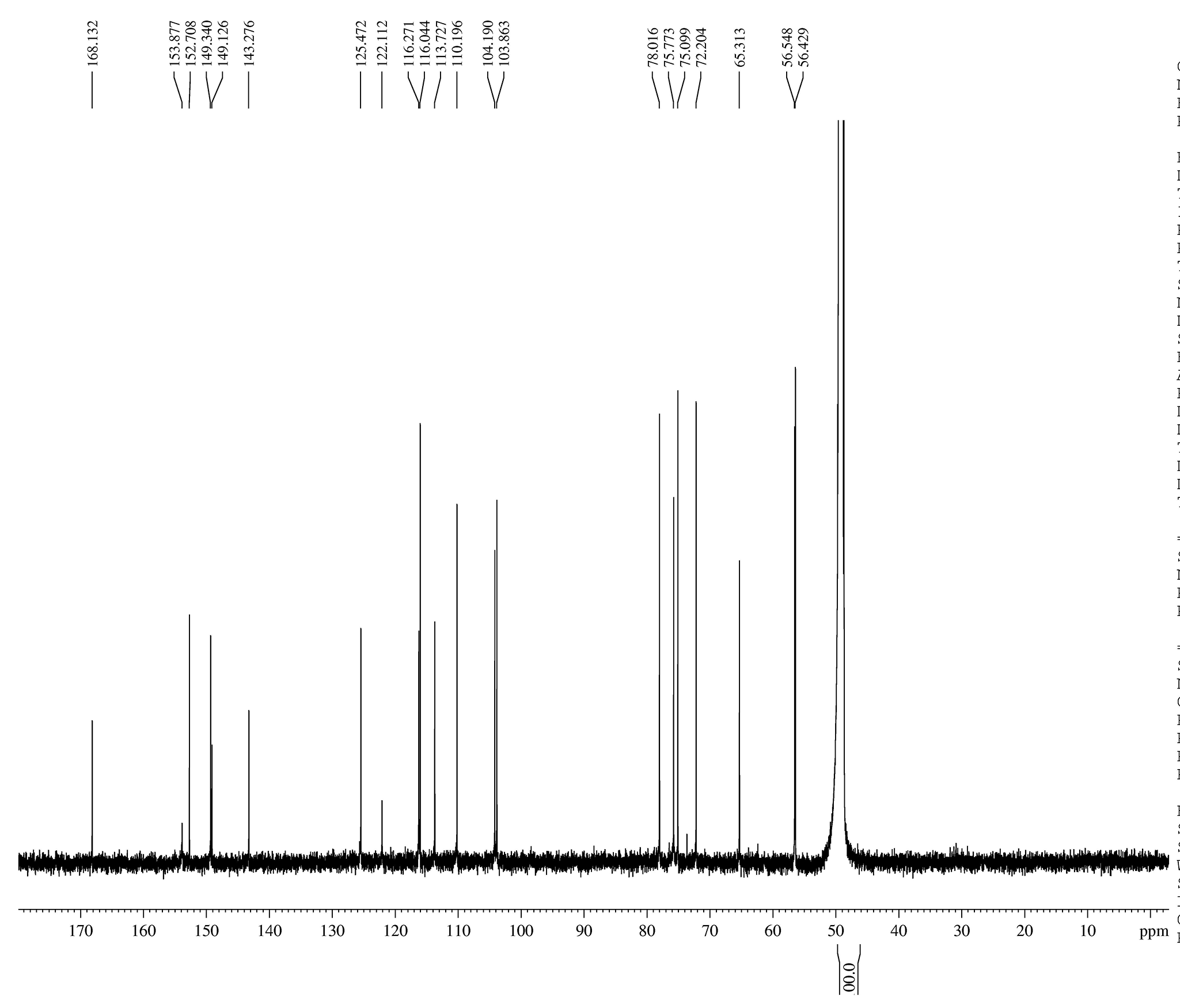

Supplement: Figure S10 — 13C NMR (150 MHz) spectrum of compound 2 in CD3OD. (TIF) [file pone.0104544.s010.tif]

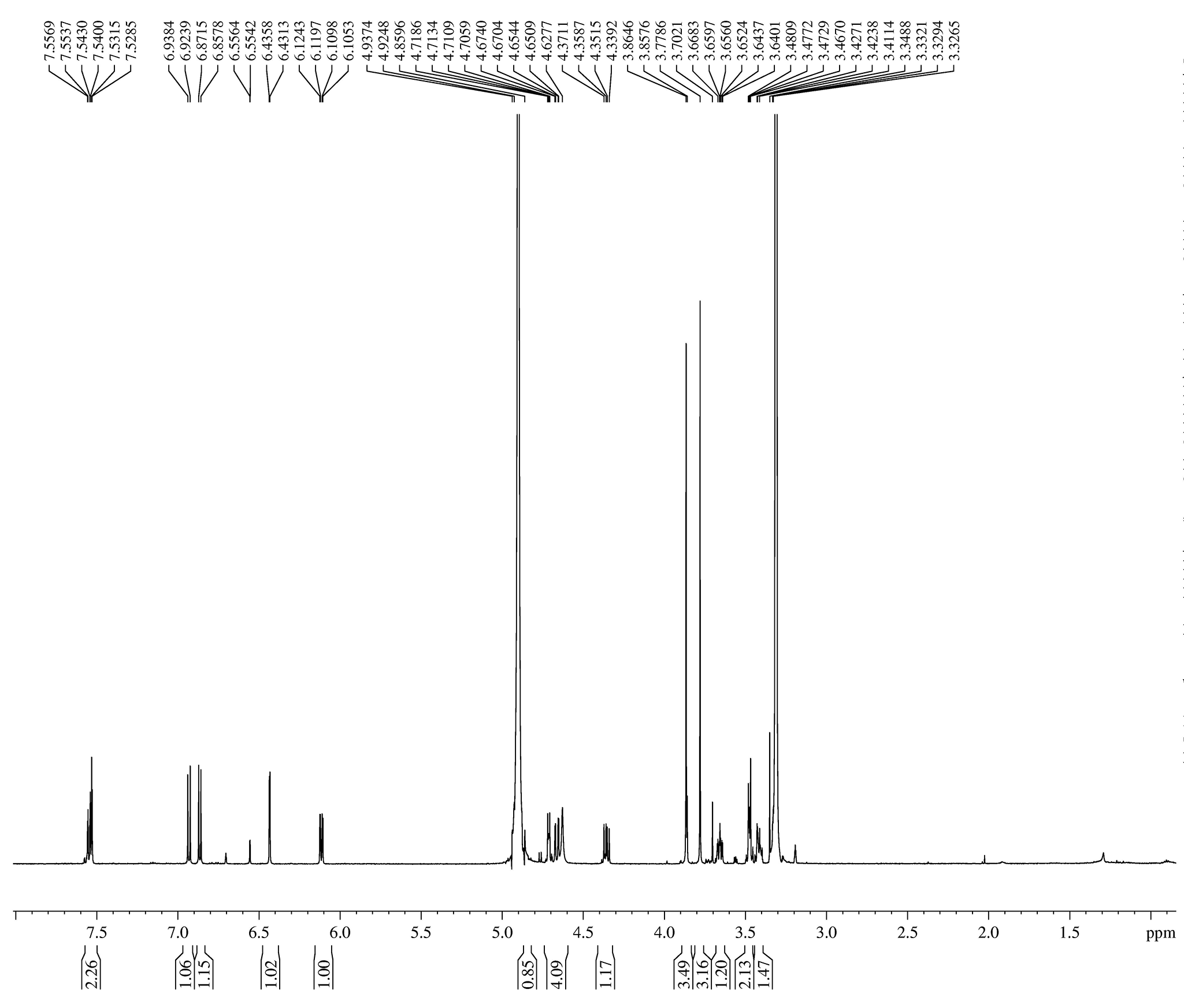

Supplement: Figure S11 — 1H NMR (600 MHz) spectrum of compound 3 in CD3OD. (TIF) [file pone.0104544.s011.tif]

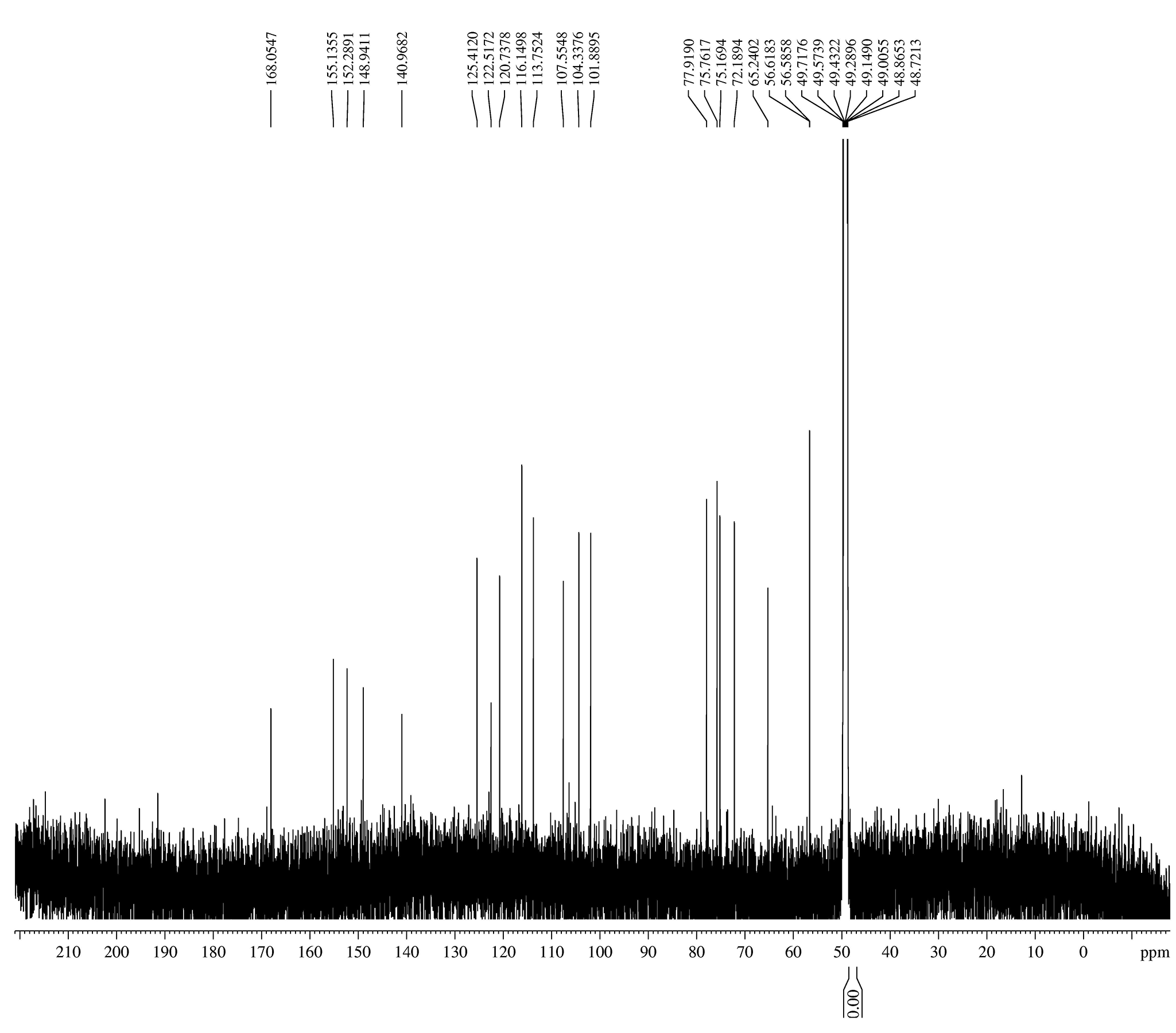

Supplement: Figure S12 — 13C NMR (150 MHz) spectrum of compound 3 in CD3OD. (TIF) [file pone.0104544.s012.tif]

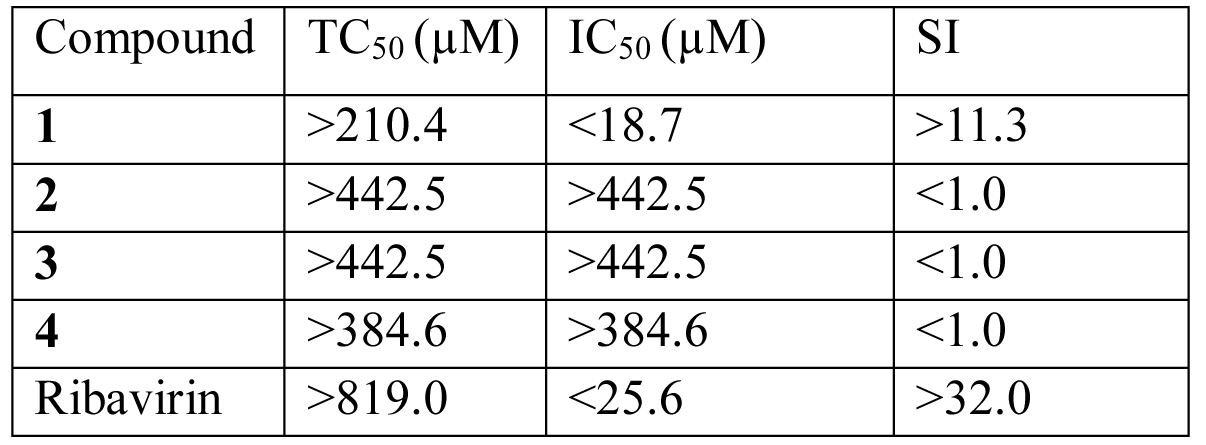

Supplement: Table S1 — Screening of compounds 1–3 and (+)-pinoresinol 4- O - β -d-glucopyranoside for inhibiting influenza viruses [A/PR/8/34 (H1N1)] activity. (TIF) [file pone.0104544.s013.tif]
